# CSF MTBR-tau243 is a specific biomarker of tau tangle pathology in Alzheimer’s disease

**DOI:** 10.1038/s41591-023-02443-z

**Published:** 2023-07-13

**Authors:** Kanta Horie, Gemma Salvadó, Nicolas R. Barthélemy, Shorena Janelidze, Yan Li, Yingxin He, Benjamin Saef, Charles D. Chen, Hong Jiang, Olof Strandberg, Alexa Pichet Binette, Sebastian Palmqvist, Chihiro Sato, Pallavi Sachdev, Akihiko Koyama, Brian A. Gordon, Tammie L. S. Benzinger, David M. Holtzman, John C. Morris, Niklas Mattsson-Carlgren, Erik Stomrud, Rik Ossenkoppele, Suzanne E. Schindler, Oskar Hansson, Randall J. Bateman

**Affiliations:** 1 The Tracy Family SILQ Center, Washington University School of Medicine, St Louis, MO USA; 2 Department of Neurology, Washington University School of Medicine, St. Louis, MO USA; 3 Eisai Inc., Nutley, NJ USA; 4 Clinical Memory Research Unit, Department of Clinical Sciences Malmö, Lund University, Lund, Sweden; 5 Department of Radiology, Washington University School of Medicine, St. Louis, MO USA; 6 Memory Clinic, Skåne University Hospital, Malmö, Sweden; 7 Knight Alzheimer Disease Research Center, Washington University School of Medicine, St. Louis, MO USA; 8 Hope Center for Neurological Disorders, Washington University School of Medicine, St. Louis, MO USA; 9 Wallenberg Center for Molecular Medicine, Lund University, Lund, Sweden; 10 Department of Neurology, Skåne University Hospital, Lund, Sweden; 11 Alzheimer Center Amsterdam, Neurology, Vrije Universiteit Amsterdam, Amsterdam UMC location VUmc, Amsterdam, The Netherlands; 12 Amsterdam Neuroscience, Neurodegeneration, Amsterdam, The Netherlands

**Keywords:** Diagnostic markers, Alzheimer's disease

## Abstract

Aggregated insoluble tau is one of two defining features of Alzheimer’s disease. Because clinical symptoms are strongly correlated with tau aggregates, drug development and clinical diagnosis need cost-effective and accessible specific fluid biomarkers of tau aggregates; however, recent studies suggest that the fluid biomarkers currently available cannot specifically track tau aggregates. We show that the microtubule-binding region (MTBR) of tau containing the residue 243 (MTBR-tau243) is a new cerebrospinal fluid (CSF) biomarker specific for insoluble tau aggregates and compared it to multiple other phosphorylated tau measures (p-tau181, p-tau205, p-tau217 and p-tau231) in two independent cohorts (BioFINDER-2, *n* = 448; and Knight Alzheimer Disease Research Center, *n* = 219). MTBR-tau243 was most strongly associated with tau-positron emission tomography (PET) and cognition, whereas showing the lowest association with amyloid-PET. In combination with p-tau205, MTBR-tau243 explained most of the total variance in tau-PET burden (0.58 ≤ *R*^2^ ≤ 0.75) and the performance in predicting cognitive measures (0.34 ≤ *R*^2^ ≤ 0.48) approached that of tau-PET (0.44 ≤ *R*^2^ ≤ 0.52). MTBR-tau243 levels longitudinally increased with insoluble tau aggregates, unlike CSF p-tau species. CSF MTBR-tau243 is a specific biomarker of tau aggregate pathology, which may be utilized in interventional trials and in the diagnosis of patients. Based on these findings, we propose to revise the A/T/(N) criteria to include MTBR-tau243 as representing insoluble tau aggregates (‘T’).

## Abstract (web summary)

CSF MTBR-tau243 is more related to tau tangles and clinical cognitive impairment in Alzheimer’s disease than phospho-tau biomarkers, which are more related to amyloid plaques.

## Main

Given the growing interest in tau-targeted therapeutics for Alzheimer’s disease (AD), there is a critical need for reliable and specific biomarkers of insoluble, aggregated tau to understand AD pathophysiology and to evaluate the effects of treatments^1^. PET with radio ligands that bind to fibrillar forms of tau reflect the burden of insoluble AD-specific tau aggregates in the brain, including neurofibrillary tangles (NFTs) and neuropil threads^2–6^. Tau-PET imaging studies have shown that insoluble tau aggregates are strongly associated with cognitive decline even during the early pre-symptomatic stages of AD^7^ and tau-PET is the most accurate prognostic marker of AD available today^8^; however, PET imaging is highly expensive and needs a complex infrastructure, which reduces its use to only highly specialized centers. In contrast, fluid biomarkers are less expensive and are more clinically accessible. The most widely used fluid biomarkers of tau are N-terminal or mid-domain total tau (t-tau) and phosphorylated tau species resulting from cleavage near residue 224 of tau^9,10^, including tau phosphorylated at residues 181, 217 and 231 (p-tau181, p-tau217 and p-tau231) (refs. ^11–17^). But, these biomarkers are strongly associated with increasing burden of amyloid plaques more than insoluble tau aggregates^18–20^. For instance, plasma and CSF concentrations of these p-tau species are already increased in preclinical AD many years before widespread insoluble tau aggregates in the neocortex are observed^21–24^. Further, recent clinical trials have demonstrated substantial reductions of CSF or plasma concentrations of t-tau, p-tau181 and p-tau217 (refs. ^25–28^) in response to anti-amyloid passive immunotherapies, which substantially remove amyloid plaques. Neuropathological and imaging studies have also reported strong associations between these fluid biomarkers and amyloid plaques^19,20,29^. In addition, animal studies have found that CSF t-tau and p-tau are increased in mouse models with amyloid β (Aβ) pathology, even when no aggregated tau pathology is observed^23,30–32^. Taken together, these findings indicate that plasma and CSF concentrations of N-terminal to mid-domain t-tau and p-tau do not directly represent insoluble tau aggregates, but rather reflect a response to amyloid plaque pathology. Thus, there is currently no fluid biomarker that specifically reflects AD-related tau pathology.

In this study, we therefore evaluated a new CSF biomarker of insoluble tau aggregates. Notably, tau species that contain MTBR-tau are a major component of insoluble tau aggregates in the brain^33–37^, but these fragments have been poorly investigated as candidate biomarkers. In an initial study, with a small sample size of controls and AD patients (*n* = 35), we showed preliminary results that MTBR-tau was present in human CSF and that a specific MTBR-tau species containing residue 243 (MTBR-tau243) was strongly associated with tau-PET and disease progression^33^. Here, we expanded these results to two large independent sporadic AD cohorts, the Swedish BioFINDER-2 study and the Charles F and Joanne Knight Alzheimer Disease Research Center (Knight ADRC), covering the whole AD continuum, with available amyloid-PET and tau-PET images. In this study, we compared the performance of MTBR-tau243 to other CSF phosphorylated tau measures, including p-tau181, p-tau205, p-tau217 and p-tau231 phosphorylation occupancies (% p-tau to total tau ratio), which are also reported as biomarkers to recapitulate AD pathologies^21,29^ and we showed that MTBR-tau243 was the fluid biomarker most strongly associated with tau-PET. We also investigated the proportion of variation in CSF biomarker levels explained by amyloid-PET and tau-PET measures of pathology. Then, we evaluated longitudinal CSF biomarker changes to investigate their rate of change based on the presence or absence of amyloid and tau pathologies to indicate which are increasing with amyloid versus tau pathologies. Finally, we assessed whether prediction of continuous AD-related measures could be improved by the combination of multiple biomarkers and found that MTBR-tau243, together with p-tau205, could optimally predict tau-PET measures and cognitive impairment.

## Results

### Participants characteristics

**Extended Data Fig. 1:**
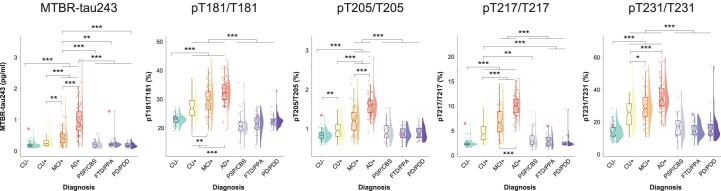
CSF biomarkers by diagnosis. Levels of each CSF biomarkers by clinical diagnosis and amyloid status in the BioFINDER-2 cohort (n = 448). Amyloid-positive participants were selected based on CSF Aβ42/40 (CSF Aβ42/40 < 0.08). Larger pink dots represent two MAPT R406W mutation carriers (one CU- and the other in FTD groups), both amyloid-negative but with substantial tau-PET binding. In boxplots, central band represents the median of the group, the lower and upper hinges correspond to the first and third quartiles, and the whiskers represent the maximum/minimum value or the 1.5 IQR from the hinge, whatever is lower). Differences in CSF biomarker levels by diagnostic groups were tested using ANCOVA adjusted for age and sex. Post hoc analyses were performed two-sided using the Tuckey test. Actual p values are reported in Supplementary Table 1 for space reasons. p < 0.050; **, p < 0.010; ***, p < 0.001. Abbreviations: AD + , Alzheimer’s disease dementia amyloid positive; CBS, corticobasal syndrome; CU-, cognitively unimpaired amyloid negative; CU + , cognitively unimpaired amyloid positive; FTD, frontotemporal dementia; MCI + , mild cognitive impairment amyloid positive; MTBR, microtubule-binding region; PD, Parkinson’s disease; PDD, Parkinson’s disease dementia; PPA, primary progressive aphasia; PSP, progressive supranuclear palsy.

The BioFINDER-2 cohort included 448 individuals, the majority of whom had cognitive impairment (281, 63%): 81 cognitively unimpaired Aβ negative (CU−), 79 cognitively unimpaired Aβ positive (CU+), 90 Aβ positive with mild cognitive impairment (MCI+), 102 Aβ positive with AD dementia (AD+) and 96 with other dementias (non-AD) (Table 1). The average age was 70.9 ± 8.4 years (mean ± s.d.), 221 (49.3%) were women and 258 (57.6%) were *APOE* ε4 carriers. The Knight ADRC cohort included 219 individuals, most of whom were cognitively unimpaired (171, 78%): 83 CU−, 88 CU+, 35 very mild AD and 13 AD+. The average age was 71.2 ± 6.6 years, 112 (51.1%) were women and 96 (43.8%) were *APOE* ε4 carriers (Extended Data Table 1). CSF biomarkers were measured in the BioFINDER-2 and the Knight ADRC cohorts, including MTBR-tau243 concentration, as well as the phosphorylation occupancy at different tau residues (percent pT181/T181, pT205/T205, pT217/T217 and pT231/T231). The phosphorylation occupancy represents the percentage of soluble tau phosphorylated at a certain amino acid position (Methods), which is a more specific measure of phosphorylation not confounded with total tau concentrations and superior to the corresponding p-tau concentration in prediction of abnormal Aβ status^29,38^. In Extended Data Fig. 1 and Supplementary Table 1, we compared the CSF levels of all biomarkers in all diagnostic groups in the BioFINDER-2 cohort. We observed that MTBR-tau243 concentrations were not increased in other non-AD tauopathies such as progressive supranuclear palsy or frontotemporal dementia (FTD), thus suggesting a high specificity for AD-related tau. Further, we did not observe any significant difference between MTBR-tau243 concentrations in CU+ compared to CU−. Of note, we found that two outliers (one in CU− and the other in FTD) that had very high levels of MTBR-tau243 were MAPT R406W mutation carriers who were amyloid negative, but clearly tau-PET positive (indicated in Extended Data Fig. 1).

**Table 1:** BioFINDER-2 participants characteristics

	Overall		CU−		CU+		MCI+		AD+		Non-AD	
	*n* =	448	*n* =	81	*n* =	79	*n* =	90	*n* =	102	*n* =	96
**Demographics**												
Age, years	448	70.9 (8.4)	81	69.9 (9.7)	79	70.5 (9.5)	90	71.7 (7.3)	102	72.5 (6.9)	96	69.8 (8.7)
Women, *n*	448	221 [49.3%]	81	40 [49.4%]	79	40 [50.6%]	90	38 [42.2%]	102	57 [55.9%]	96	46 [47.9%]
*APOE-ε4* carriers, *n*	447	258 [57.6%]	81	27 [33.3%]	79	58 [73.4%]	89	65 [72.2%]	102	76 [74.5%]	96	32 [33.3%]
Years of education	443	12.2 (3.7)	81	12.0 (3.2)	79	12.2 (3.4)	89	12.5 (4.6)	101	11.9 (3.9)	93	12.5 (3.5)
**CSF Aβ measures**												
CSF Aβ42/40	427	0.0687 (0.0293)	81	0.1080 (0.0132)	79	0.0553 (0.0134)	85	0.0492 (0.0130)	100	0.0447 (0.0113)	82	0.0922 (0.0217)
CSF Aβ42/40 positivity, *n*	427	290 [64.7%]	81	0 [0%]	79	79 [100%]	90	90 [100%]	102	102 [100%]	82	26 [27.1%]
**Amyloid-PET and Tau-PET measures**												
Amyloid-PET, centiloids	268	38.4 (44.4)	81	−4.5 (9.3)	79	41.6 (36.5)	88	71.6 (35.0)	7	115.0 (23.3)	11	12.6 (25.1)
Amyloid-PET positivity, *n*	268	148 [33.0%]	81	2 [2.5%]	79	55 [69.6%]	88	79 [87.8%]	7	7 [6.9%]	11	3 [3.1%]
Tau-PET Braak I–IV, SUVR	443	1.53 (0.61)	81	1.17 (0.09)	79	1.23 (0.21)	90	1.51 (0.45)	101	2.40 (0.60)	92	1.17 (0.12)
Tau-PET positivity, *n*	443	162 [36.2%]	81	1 [1.2%]	79	11 [13.9%]	90	45 [50.0%]	101	101 [99.0%]	92	4 [4.2%]
**CSF tau by mass spectrometry**												
pT181/T181 (%)	448	26.9 (5.6)	81	22.8 (1.6)	79	27.2 (3.8)	90	29.5 (4.9)	102	32.6 (4.1)	96	21.8 (3.6)
pT205/T205 (%)	448	1.14 (0.45)	81	0.79 (0.14)	79	0.97 (0.28)	90	1.24 (0.41)	102	1.70 (0.32)	96	0.86 (0.24)
pT217/T217 (%)	448	6.56 (4.12)	81	2.78 (0.83)	79	5.71 (2.51)	90	8.02 (3.24)	102	11.90 (2.53)	96	3.44 (1.70)
pT231/T231 (%)	448	12.30 (5.69)	81	7.09 (2.15)	79	12.70 (4.16)	90	14.80 (4.59)	102	17.90 (4.46)	96	8.14 (3.61)
MTBR-tau243 (pg/ml)	448	0.445 (0.424)	81	0.192 (0.089)	79	0.281 (0.165)	90	0.449 (0.279)	102	0.992 (0.502)	96	0.207 (0.139)
**Cognitive measures**												
MMSE	447	25.8 (4.6)	81	29.1 (1.1)	79	28.8 (1.3)	90	26.8 (1.9)	101	19.8 (4.4)	96	25.8 (4.0)

Data are presented as mean (s.d.). Values in square brackets indicate the % in total number within the group. SUVR, standardized uptake value ratio.

### Association between CSF marker and amyloid or tau measure

**Fig. 1:**
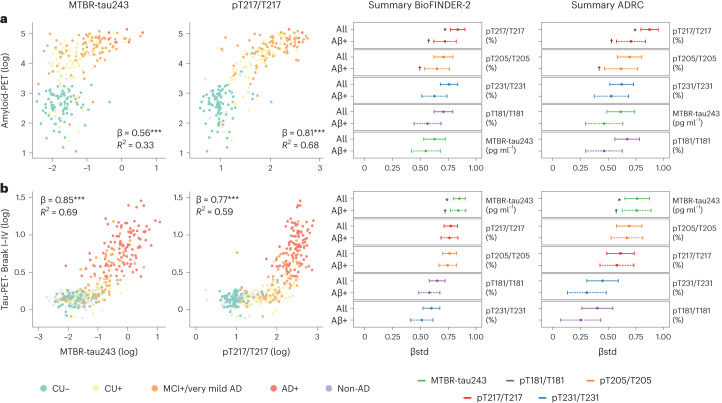
Associations between CSF biomarkers and amyloid-PET and tau-PET. **a**,**b**, Associations between CSF biomarkers and amyloid-PET (**a**) and tau-PET (**b**). First two columns show scatter-plots of MTBR-tau243 (first column) and pT217/T217 (second column) and amyloid-PET (*n* = 268) or tau-PET (*n* = 443) in BioFINDER-2 participants, color-coded by diagnosis and amyloid status. Linear regression models, adjusting for age and sex, were used to obtain β, *P* values (asterisks) and *R*^2^ shown in the plots. Scatter-plots for all the biomarkers in both cohorts are shown in Extended Data Figs. 1 and 2. The third and fourth columns show standardized β (βstd) of the association between each CSF biomarker and amyloid- or tau-PET in BioFINDER-2 and Knight ADRC participants (*n* = 219; except for pT231/T231 in which *n* = 184 for all cases), respectively. Solid and dashed lines show standardized β (central dot) and 95% CI when all participants or only amyloid-positive participants (BioFINDER-2, amyloid-PET, *n* = 172, tau-PET, *n* = 287; Knight ADRC, *n* = 136; except for pT231/T231 in which *n* = 117) were included, respectively. Asterisks (crosses) show the highest or not significantly different standardized β in all (amyloid-positive only) participants, in each cohort and outcome based on bootstrapping. Thus, those biomarkers without asterisks or crosses have statistically weaker correlations. Aβ-positive participants were selected based on CSF Aβ42/40 previously validated cutoff values (CSF Aβ42/40 < 0.08 in BioFINDER-2 and CSF Aβ42/40 < 0.0673 in Knight ADRC). Association *P* values were derived from two-sided tests and bootstrapping *P* values were obtained from one-sided tests, all without adjustment for multiple comparisons. All *P* values from associations between CSF biomarkers and amyloid-PET and tau-PET were <0.001.

**Extended Data Fig. 2:**
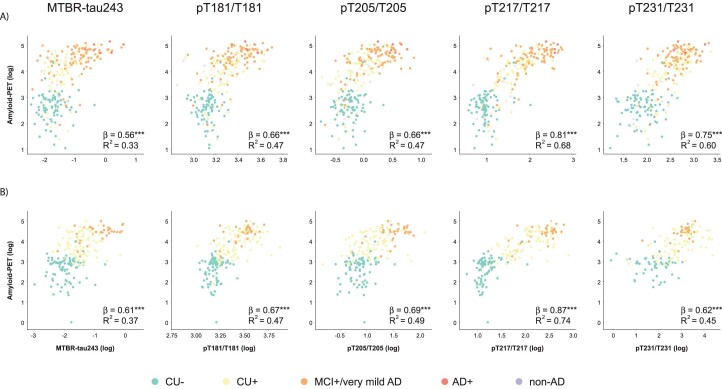
Associations between all CSF biomarkers and Aβ-PET. Associations between CSF biomarkers and amyloid-PET (Centiloid) in BioFINDER-2 (A) and Knight ADRC (B) participants. Linear regression models, adjusting for age and sex, were used to obtain standardized β and p values shown in the plots. In the BioFINDER-2 cohort, orange dots represent MCI+ participants whereas in the Kinght-ADRC cohort, represent very mild AD.  *, p < 0.050; **, p < 0.010; ***, p < 0.001. Abbreviations: AD + , Alzheimer’s disease dementia amyloid positive; CL, Centiloids, CSF, cerebrospinal fluid; CU-, cognitively unimpaired amyloid negative; CU + , cognitively unimpaired amyloid positive; MCI + , mild cognitive impairment amyloid positive; MTBR, microtubule binding region; non-AD, non-Alzheimer’s disease dementia; PET, positron emission tomography.

**Extended Data Fig. 3:**
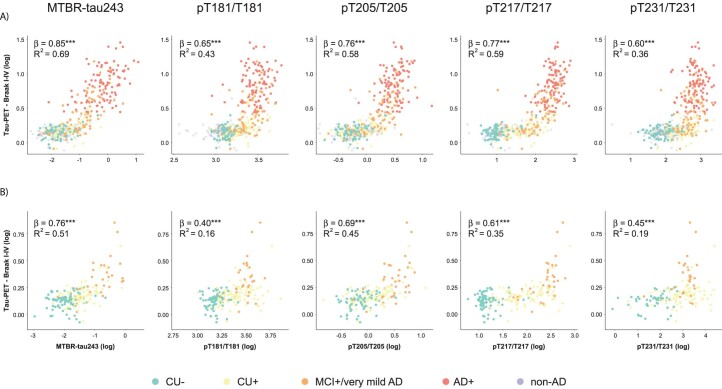
Associations between all CSF biomarkers and tau-PET in the Braak I-IV ROI. Associations between CSF biomarkers and tau-PET in the Braak I-IV ROI (SUVR) in BioFINDER-2 (A) and Knight ADRC (B) participants. Linear regression models, adjusting for age and sex, were used to obtain standardized β and p-values shown in the plots. In the BioFINDER-2 cohort, orange dots represent MCI+ participants whereas in the Kinght-ADRC cohort, represent very mild AD. *, p < 0.050; **, p < 0.010; ***, p < 0.001. Abbreviations: AD + , Alzheimer’s disease dementia amyloid positive; CSF, cerebrospinal fluid; CU-, cognitively unimpaired amyloid negative; CU + , cognitively unimpaired amyloid positive; MCI + , mild cognitive impairment amyloid positive; MTBR, microtubule binding region; non-AD, non-Alzheimer’s disease dementia; PET, positron emission tomography, SUVR, standardized uptake value ratio.

CSF MTBR-tau243, pT181/T181, pT205/T205, pT217/T217 and pT231/T231 were assessed for association with amyloid-PET and tau-PET measures of pathology using linear regression models adjusting for age and sex. All participants were compared, in addition to the amyloid-positive-only subgroup, to separate out amyloid from tau pathology effects (Fig. 1). The phosphorylation occupancy at T217 (pT217/T217) was the CSF measure most strongly correlated with amyloid-PET (BioFINDER-2, β = 0.81, 95% confidence interval (CI) 0.74–0.88; Knight ADRC, β = 0.87, 0.79–0.95; all *P* < 0.001; Fig. 1a and Extended Data Table 2). MTBR-tau243 concentration was the CSF measure most strongly associated with tau-PET in all participants (BioFINDER-2, β = 0.85, 0.80–0.90; Knight ADRC, β = 0.76, 0.65–0.87; all *P* < 0.001) and in amyloid-positive participants (BioFINDER-2, β = 0.84, 0.77–0.91; Knight ADRC, β = 0.76, 0.63–0.89; all *P* < 0.001; Fig. 1b and Extended Data Table 2). Notably, the CSF MTBR-tau243 concentration was significantly more strongly associated with tau-PET when compared to pT217/T217 (BioFINDER-2, β = 0.77, 0.71–0.83, *P*_comp_ < 0.001; Knight ADRC, β = 0.61, 0.49–0.73, *P*_comp_ < 0.001) in all participants and in amyloid-positive particip = ants (BioFINDER-2, β0.76, 0.69–0.84, *P*_comp_ = 0.001; Knight ADRC, β = 0.58, 0.43–0.73, *P*_comp_ = 0.001; Extended Data Table 2). Scatter-plots for the associations of all CSF tau biomarkers and amyloid-PET and tau-PET in both cohorts are shown in Extended Data Figs. 2 and 3, respectively.

We also investigated correlations of CSF tau measures with CSF Aβ42/40. Of the CSF tau measures, pT217/T217 was most strongly correlated with CSF Aβ42/40 (BioFINDER-2, β = −0.80, 95% CI −0.86 to −0.74; Knight ADRC, β = −0.88, −0.95 to −0.81; all *P* < 0.001; Supplementary Fig. 1 and Supplementary Table 2). In contrast, MTBR-tau243 concentration showed significantly lower association with CSF Aβ42/40 compared to pT217/T217 (BioFINDER-2, β = −0.63, −0.70 to −0.55, *P*_comp_ < 0.001; Knight ADRC, β = −0.59, −0.71 to −0.47; all *P* < 0.001; Supplementary Fig. 1 and Supplementary Table 2).

**Extended Data Fig. 4:**
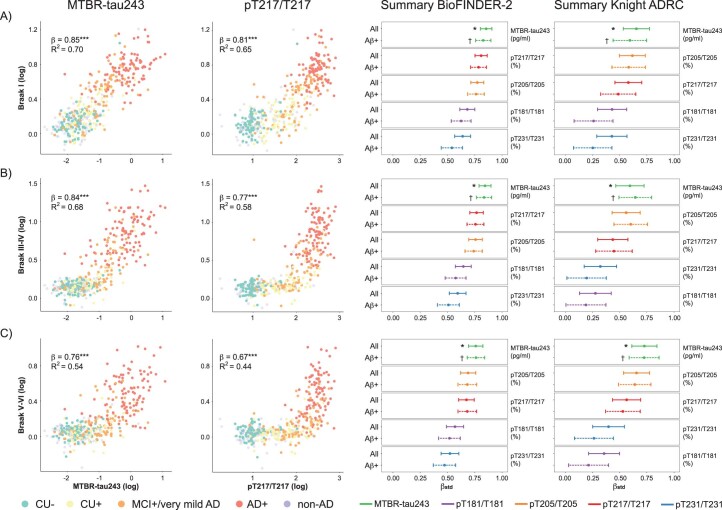
Associations between CSF biomarkers and tau-PET in different Braak regions. Associations between CSF biomarkers and tau-PET in Braak I (A), Braak III–IV (B) and Braak V–VI (C) regions. First two columns show scatter-plots of MTBR-tau243 (first column) and pT217/T217 (second column) and tau-PET in BioFINDER-2 participants (n = 443), colored by diagnosis and amyloid status. Linear regression models, adjusting for age and sex, were used to obtain standardized β, p-values (asterisks) and R^2^ shown in the plots. Third and fourth columns show standardized β of the association between each CSF biomarker and tau-PET in BioFINDER-2 and Knight ADRC participants (n = 219; except for pT231/T231 in which n = 184), respectively. Solid and dashed lines show standardized β (central dot) and 95%CI when all participants or only amyloid positive participants (BioFINDER-2: n = 287; Knight ADRC, n = 136) were included, respectively. Asterisks (crosses) show the highest or not significantly different standardized β in all (amyloid positive only) participants, in each cohort and outcome. Amyloid-positive participants were selected based on CSF Aβ42/40 previously validated cutoffs (CSF Aβ42/40 < 0.08 in BioFINDER-2 and CSF Aβ42/40 < 0.0673 in Knight ADRC). Association p-values were based on two-sided tests and bootstrapping p-values from one-sided tests, all unadjusted for multiple comparisons. All p-values from associations between CSF biomarkers and tau-PET shown in the scatter-plots were <0.001. *, p < 0.050; **, p < 0.010; ***, p < 0.001. Abbreviations: Aβ + , amyloid positive; AD + , Alzheimer’s disease dementia amyloid positive; CSF, cerebrospinal fluid; CU-, cognitively unimpaired amyloid negative; CU + , cognitively unimpaired amyloid positive; MCI + , mild cognitive impairment amyloid positive; MTBR, microtubule binding region; non-AD, non-Alzheimer’s disease dementia; PET, positron emission tomography; SUVR, standardized uptake value ratio.

Correlations of CSF tau measures and tau-PET signal in different Braak regions (entorhinal (Braak I), temporal (Braak III–IV) and neocortical (Braak V–VI)) were also investigated as an additional analysis. Comparisons in the amyloid-positive only group demonstrated that CSF MTBR-tau243 had the highest correlations with all Braak regions (BioFINDER-2, β = 0.85, 0.84 and 0.76; Knight ADRC, β = 0.83, 0.84 and 0.76 for each Braak regions, respectively; all *P* < 0.001; Extended Data Fig. 4 and Supplementary Table 3).

### Biomarker variation explained by amyloid and tau pathologies

**Fig. 2:**
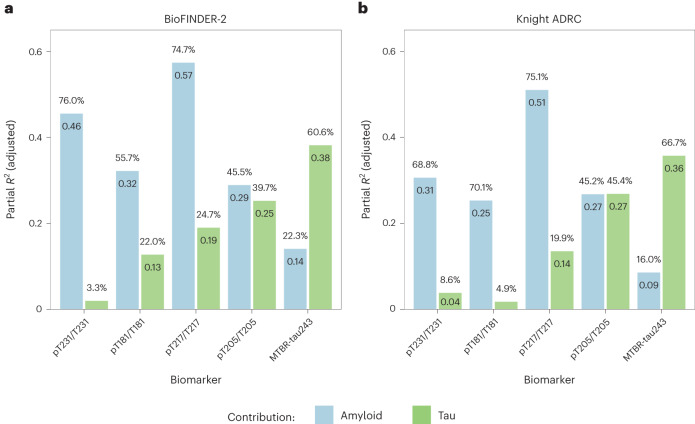
Proportion of variation of CSF biomarker levels explained by amyloid-PET and tau-PET. **a**,**b**, Partial *R*^2^ values are displayed within the columns and the percentages of partial *R*^2^ over the total *R*^2^ of the model are indicated above each column for BioFINDER-2 (**a**) and Knight ADRC participants (**b**). These values were computed using individual CSF biomarkers as outcomes and amyloid and tau measures as predictors in linear regression models adjusted for age and sex, within each CSF biomarker and cohort. The percentages may not sum up to 100% due to potential shared variance. The biomarkers are arranged from left to right based on the increasing contribution (%) of tau to their levels.

Next, we evaluated the proportion of variation in CSF biomarkers explained by amyloid and tau pathologies. CSF biomarker levels were included as the outcome and amyloid-PET and tau-PET were both included as predictors controlling for age and sex, in our models. In the BioFINDER-2 cohort, variance in CSF pT217/T217 levels was significantly better explained by Aβ pathology as assessed with amyloid-PET, than tau (Aβ, partial *R*^2^ (p*R*^2^) = 0.57, 74.7% *R*^2^ versus tau, p*R*^2^ = 0.19, 24.7% *R*^2^, *P*_*comp*_ < 0.001; Fig. 2a and Extended Data Table 3). CSF pT231/T231 (Aβ, p*R*^2^ = 0.46, 76.0% *R*^2^; tau, p*R*^2^ = 0.02, 3.3% *R*^2^, *P*_*comp*_ < 0.001) and pT181/T181 (Aβ, p*R*^2^ = 0.32, 55.7% *R*^2^; tau, p*R*^2^ = 0.13, 22.0% *R*^2^, *P*_*comp*_ = 0.006) were also significantly better explained by Aβ pathology. In contrast, variance in CSF MTBR-tau243 concentrations were significantly better explained by tau pathology (Aβ, p*R*^2^ = 0.14, 22.3% *R*^2^; tau, p*R*^2^ = 0.38, 60.6% *R*^2^, *P*_*comp*_ < 0.001). The contribution of tau and amyloid to CSF pT205/T205 was similar, with the difference between both being non-significant (Aβ, p*R*^2^ = 0.29, 45.4% *R*^2^; tau, p*R*^2^ = 0.25, 39.7% *R*^2^, *P*_*comp*_ = 0.657).

Similar trends were observed in the Knight ADRC cohort, although with a greater proportion of variance was explained by Aβ pathology for all CSF biomarkers, likely because this cohort included relatively few individuals with substantial tau pathology (only *n* = 36 (16.4%) were tau-PET positive). CSF pT217/T217 (Aβ, p*R*^2^ = 0.51, 75.1% *R*^2^; tau, p*R*^2^ = 0.14, 19.9% *R*^2^, *P*_*comp*_ < 0.001), pT181/T181 (Aβ, p*R*^2^ = 0.25, 70.1% *R*^2^; tau, p*R*^2^ = 0.02, 4.9% *R*^2^, *P*_*comp*_ < 0.001) and pT231/T231 (Aβ, p*R*^2^ = 0.31, 68.8% *R*^2^; tau, p*R*^2^ = 0.04, 8.6% *R*^2^, *P*_*comp*_ < 0.001) were better explained by Aβ pathology. In contrast, tau pathology was the major contributor on explaining variance in CSF MTBR-tau243 levels (Aβ, p*R*^2^ = 0.09, 16.0% *R*^2^; tau, p*R*^2^ = 0.36, 66.7% *R*^2^, *P*_*comp*_ < 0.001). Of note, pT205/T205 levels were explained similarly by both tau and amyloid (Aβ, p*R*^2^ = 0.27, 45.2% *R*^2^; tau, p*R*^2^ = 0.27, 45.4% *R*^2^, *P*_*comp*_ = 0.990; Fig. 2b and Extended Data Table 3).

Because dementia patients of BioFINDER-2 did not undergo amyloid-PET, analyses were repeated in both cohorts with all participants using CSF Aβ42/40 rather than amyloid-PET as the measure of Aβ pathology (Supplementary Fig. 2 and Extended Data Table 3). Levels of CSF pT217/T217 were slightly, but significantly better explained by CSF Aβ42/40 levels than tau-PET (BioFINDER-2, 66.6% *R*^2^ versus 56.5%, *P* = 0.044; Knight ADRC, 87.6% *R*^2^ versus 28.9%, *P*_*comp*_ < 0.001). CSF Aβ42/40 continued to be the major factor associated with CSF pT231/T231 (BioFINDER-2, 69.8% versus 19.1%, *P*_*comp*_ < 0.001; Knight ADRC, 84.0% versus 7.0%, *P*_*comp*_ < 0.001) and pT181/T181 (BioFINDER-2, 52.9% versus 33.7, *P*_*comp*_ = 0.014; Knight ADRC, 82.8% versus 3.5%, *P*_*comp*_ < 0.001). In these models, tau pathology remained the major factor explaining variance in MTBR-tau243 levels (BioFINDER-2, 21.4% versus 75.1%, *P*_*comp*_ < 0.001; Knight ADRC, 33.5% versus 63.8%, *P*_*comp*_ = 0.014). For pT205/T205, the major contributor was tau pathology (BioFINDER-2, 19.6% versus 66.8%, *P*_*comp*_ < 0.001) although the difference was not significant in Knight ADRC (36.5% versus 57.6%, *P*_*comp*_ = 0.125).

### Longitudinal change in CSF biomarkers

**Fig. 3:**
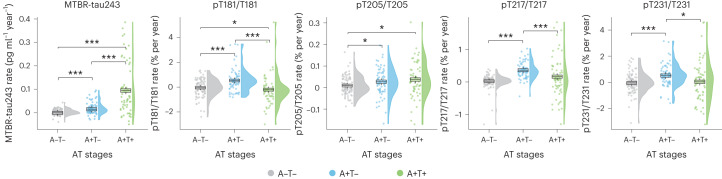
Longitudinal CSF biomarkers change by baseline amyloid and tau status. Rates of change in CSF biomarkers per baseline amyloid (A) and tau (T) status are depicted (pT231/T231: *n* = 218, rest: *n* = 220). Individual rates of change are represented by dots. Trajectories for each group are displayed as boxplots, which were generated using linear mixed models (the central band represents the median, the lower and upper hinges correspond to the first and third quartiles and the whiskers depict the maximum/minimum value or 1.5 × interquartile range from the hinge, whichever is lower). Differences among all groups were assessed using Kruskal–Wallis tests and pairwise Wilcoxon tests were employed for post hoc comparisons. Asterisks indicate the *P* values from two-sided tests without correction for multiple comparisons. Longitudinal CSF data was available only in BioFINDER-2. Amyloid-positive participants were identified using a previously validated cutoff for CSF Aβ42/40 (CSF Aβ42/40 < 0.08). Tau positivity was determined based on tau-PET SUVR in the meta-ROI (Braak I-IV, SUVR > 1.32). ROI, region of interest. The actual *P* values for A−T− versus A+T− were *P* = 0.011 (pT205/T205); for A−T− versus A+T+ were *P* = 0.011 (pT181/T181), *P* = 0.014 (pT205/T205), *P* = 0.617 (pT217/T217) and *P* = 0.980 (pT231/T231); and for A+T− versus A+T+ were *P* = 0.788 (pT205/T205), *P* = 0.007 (pT231/T231). All other comparisons yielded *P* < 0.001. *P < 0.050; **P < 0.010; ***P < 0.001.

**Extended Data Fig. 5:**
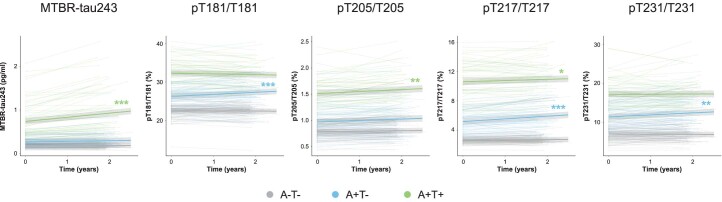
Longitudinal CSF biomarkers trajectories by baseline “A” and “T” status. Individual (shaded lines) and group (bold lines) CSF biomarker levels trajectories over time based on their A/T baseline status. Statistical differences to the reference group (A-T-) are shown with asterisks with the appropriate color. Longitudinal CSF data was only available in BioFINDER-2. Amyloid-positive participants were selected based on a CSF Aβ42/40 previously validated cutoff (CSF Aβ42/40 < 0.08). Tau positivity was assessed based on tau-PET SUVR in the meta-ROI (Braak I-IV SUVR > 1.32). *, p < 0.050; **, p < 0.010; ***, p < 0.001. Abbreviations: A-T-, amyloid and tau negative; A + T-, amyloid positive and tau negative; A + T + , amyloid positive, tau positive; CSF, cerebrospinal fluid; MTBR, microtubule binding region; PET, positron emission tomography.

Longitudinal data from the BioFINDER-2 cohort was used to examine changes in CSF biomarkers stratified by amyloid (A) and tau (T) pathology status (+ and −). Characteristics of the 220 participants with longitudinal CSF measurements are described in Supplementary Table 4. Linear mixed models were used to compare CSF longitudinal trajectories among groups (A−/T−, A+/T− and A+/T+) using post hoc pairwise Wilcoxon test when the interaction with time was significant. Amyloid status was derived from CSF Aβ42/40 levels and tau was dichotomized from tau-PET measures. Individual and group trajectories over time are shown in Extended Data Fig. 5. CSF pT217/T217, pT181/T181 and pT231/T231 had their greatest rate of increase in the A+T− group and a lower rate of increase in the A+T+ group, indicating that the rate of increase of these biomarkers was plateauing at later stages of disease when tau pathology was increasing most (Fig. 3). In contrast, CSF pT205/T205 and MTBR-tau243 had their greatest rate of increase in the A+T+ group, corresponding to matching increases in tau pathology. Notably, CSF MTBR-tau243 was increasing faster in the A+T+ group than the A+T− group (versus A−T−, Cohen’s *d* = 1.48, *P* < 0.001; versus A+T−, Cohen’s *d* = 1.13, *P* < 0.001), whereas the rate of increase in pT205/T205 was not significantly different in the A+T+ and A+T− groups (Cohen’s *d* = 0.08, *P* = 0.788; Fig. 3 and Supplementary Table 5). This suggests that CSF MTBR-tau243 would best reflect AD progression in tau-PET positive individuals.

As a sensitivity analysis, we repeated this analysis using amyloid-PET rather than CSF Aβ42/40 for classifying participants, using a previously validated threshold^39^. We found that the longitudinal trajectories for all CSF biomarkers were replicated, with pT205/T205, but especially MTBR-tau243, rates of change increasing with progressing A/T status and the rest of biomarkers having the highest rate of change at A+T− status (Supplementary Fig. 3 and Supplementary Table 5).

### Association of CSF and PET biomarkers with MMSE scores

**Fig. 4:**
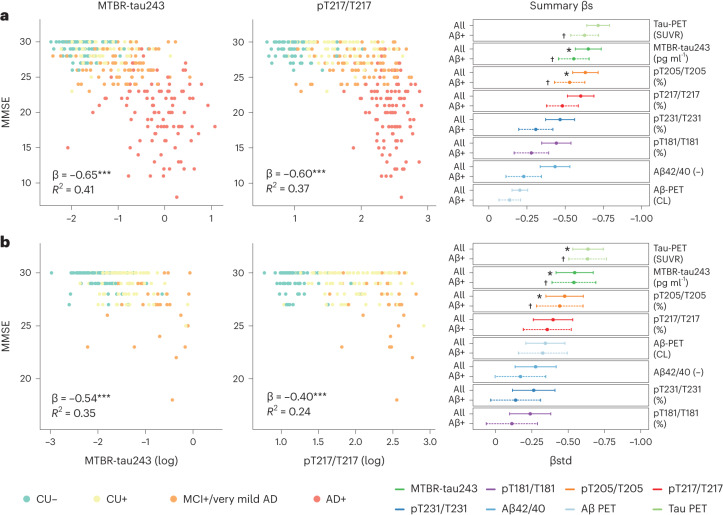
Associations between CSF biomarkers and MMSE. **a**,**b**, Associations between CSF biomarkers and MMSE are depicted for BioFINDER-2 (**a**, *n* = 342) and Knight ADRC (**b**, pT231/T231: *n* = 184, rest: *n* = 219) participants. The first two columns display scatter-plots of MTBR-tau243 (first column) and pT217/T217 (second column) against MMSE, color-coded by diagnosis and amyloid status. In the BioFINDER-2 cohort, orange dots represent MCI+ participants, while in the Knight ADRC cohort, they represent individuals with very mild AD. Linear regression models, adjusted for age, sex and years of education were utilized to obtain β coefficients, *P* values (asterisks) and *R*^2^ values shown in the plots. Scatter-plots for all biomarkers in both cohorts can be found in Extended Data Fig. 4. The third column shows the standardized β coefficients for all biomarkers, along with the associations of amyloid-PET and CSF Aβ42/40 (reversed) and tau-PET for comparison. Solid and dashed lines represent the standardized β coefficients (central dot) and 95% CI when including all participants or only amyloid-positive participants (BioFINDER-2, *n* = 261; Knight ADRC, *n* = 136, except for pT231/T231, where *n* = 117), respectively. Asterisks (crosses) indicate the highest or not significantly different standardized β coefficients in all (amyloid-positive only) participants within each cohort and outcome, based on bootstrapping. Non-AD participants from BioFINDER-2 were excluded from these analyses. Amyloid-positive participants were selected using previously validated cutoffs for CSF Aβ42/40 (CSF Aβ42/40 < 0.08 in BioFINDER-2 and CSF Aβ42/40 < 0.0673 in Knight ADRC). Association *P* values were derived from two-sided tests and bootstrapping *p* values were obtained from one-sided tests, all without adjustment for multiple comparisons. All p values for associations between CSF biomarkers and MMSE were <0.001.

**Extended Data Fig. 6:**
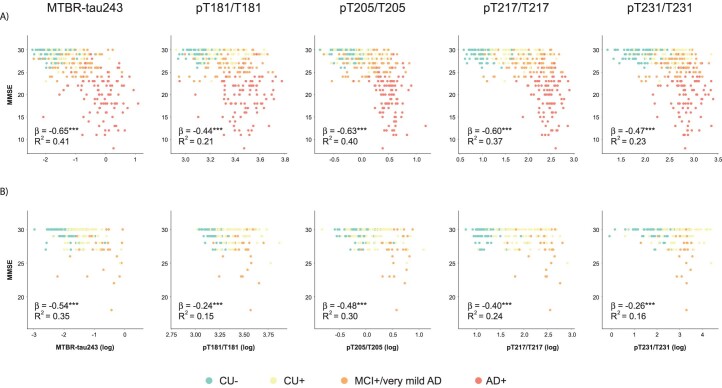
Associations between all CSF biomarkers and MMSE. In BioFINDER-2 cohort (A), orange dots represent MCI+ participants whereas in the Kinght-ADRC cohort (B), represent very mild AD. Linear regression models, adjusting for age, sex and years of education were used to obtain standardized β and p-values shown in the plots. *, p < 0.50; **, p < 0.010; ***, p <0.001. Abbreviations: AD + , Alzheimer’s disease dementia amyloid positive; CSF, cerebrospinal fluid; CU-, cognitively unimpaired amyloid negative; CU + , cognitively unimpaired amyloid positive; MCI + , mild cognitive impairment amyloid positive; MMSE, Mini Mental State Examination; MTBR, microtubule binding region; PET, positron emission tomography, SD, standard deviation; SUVR, standardized uptake value ratio.

We assessed associations of CSF and PET biomarkers with a common clinical assessment of dementia, the Mini Mental State Examination (MMSE)^40^, which was assessed in both cohorts, using linear regression models that adjusted for age, sex and years of education. MTBR-tau243 was the CSF biomarker most strongly associated with MMSE scores in all participants (BioFINDER-2, β = −0.65, −0.74 to −0.57; Knight ADRC, β = −0.54, −0.67 to −0.42, all *P* < 0.001) and amyloid-positive participants (BioFINDER-2, β = −0.56, −0.66 to −0.46; Knight ADRC, β = −0.54, −0.69 to −0.39, all *P* < 0.001; Fig. 4 and Supplementary Table 6). These associations were significantly stronger than those of pT217/T217, as assessed by bootstrapping, for all participants (BioFINDER-2, β = −0.60, −0.69 to −0.52, *P*_comp_ = 0.001; Knight ADRC, β = −0.40, −0.53 to −0.26, *P*_comp_ = 0.003) and amyloid-positive participants (BioFINDER-2, β = −0.48, −0.59 to −0.38, *P*_comp_ = 0.002; Knight ADRC, β = −0.36, −0.52 to −0.19, *P*_comp_ = 0.001); however, tau-PET was more strongly associated with MMSE than any CSF biomarker (BioFINDER-2, β = −0.73, −0.79 to −0.64, *P*_comp_ = 0.036; Knight ADRC, β = −0.64, −0.74 to −0.53, *P*_comp_ <0.001). Scatter-plots for each CSF biomarker are shown in Extended Data Fig. 6.

### Best predictors of AD-related measures and cognitive function

Finally, we aimed to determine whether combinations of CSF biomarkers could be used as accurate quantitative surrogates for amyloid-PET, tau-PET or cognitive measures. We first evaluated the variance explained by each individual biomarker for each outcome. Next, we used the least absolute shrinkage and selection operator (LASSO) procedure to select which combination of CSF biomarkers were optimal for each outcome and then, we compared this new model to the ones from the individual biomarkers.

**Fig. 5:**
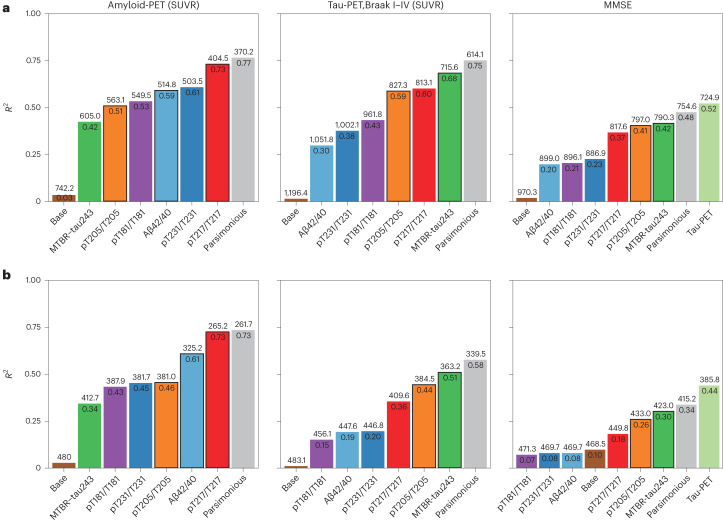
Predicting quantitative amyloid-PET, tau-PET and MMSE continuous measures with CSF biomarkers. **a**,**b**, Linear regression models were employed to predict amyloid-PET (first column, BioFINDER-2, *n* = 256), tau-PET (second column, BioFINDER-2, *n* = 422) and cognition (MMSE, third column, BioFINDER-2, *n* = 342) in BioFINDER-2 (**a**) and Knight ADRC (**b**, *n* = 184). The base model included age and sex (and years of education for MMSE) as predictors. The parsimonious model was derived by using LASSO regression to identify the optimal combination of CSF biomarkers and demographic factors (age, sex and/or years of education). Biomarkers included in the parsimonious models are indicated by a black border and their names are shown in bold. The other models solely employed individual CSF biomarkers as predictors. For comparison, CSF Aβ42/40 and tau-PET were used as predictors in independent models for predicting all outcomes and cognition only, respectively. Model comparisons were conducted using an *F*-test for nested models or Vuong’s test for non-nested models. Non-AD cases were excluded from the BioFINDER-2 cohort for the cognition analyses.

CSF pT217/T217 was the individual biomarker that best predicted amyloid-PET (BioFINDER-2, *R*^2^ = 0.73, corrected Akaike information criterion (AICc) = 404.5; Knight ADRC, *R*^2^ = 0.73, AICc = 265.2; Fig. 5 and Supplementary Table 7). Based on the LASSO regressions, we found that combining CSF pT217/T217 with pT205/T205 and Aβ42/40 significantly improved prediction of amyloid-PET in both cohorts (BioFINDER-2, *R*^2^ = 0.77, AICc = 370.2, *F* = 20.974, *P* < 0.001; Knight ADRC, *R*^2^ = 0.73, AICc = 261.7, *F* = 5.266, *P* = 0.006; Extended Data Table 4).

MTBR-tau243 was the individual biomarker that best predicted quantitative tau-PET (BioFINDER-2, *R*^2^ = 0.68, AICc = 715.6; Knight ADRC, *R*^2^ = 0.51, AICc = 363.2; Fig. 5 and Supplementary Table 7). The optimal model, which combined MTBR-tau243 and pT205/T205, significantly improved prediction of tau-PET amounts in both cohorts (BioFINDER-2, *R*^2^ = 0.75, AICc = 614.1, *F* = 116.49, *P* < 0.001; Knight ADRC, *R*^2^ = 0.58, AICc = 339.5, *F* = 30.268, *P* < 0.001; Fig. 5 and Extended Data Table 4).

MTBR-tau243 was the individual biomarker that best predicted MMSE scores (BioFINDER-2, *R*^2^ = 0.42, AICc = 790.3; Knight ADRC, *R*^2^ = 0.30, AICc = 423.0; Fig. 5 and Supplementary Table 7) and prediction improved when pT205/T205 was added (BioFINDER-2, *R*^2^ = 0.48, AICc = 754.6, *F* = 27.693, *P* < 0.001; Knight ADRC, *R*^2^ = 0.34, AICc = 415.2; Fig. 5 and Extended Data Table 4). Tau-PET predicted MMSE scores better than a combination of CSF biomarkers, but the difference was not statistically significant (BioFINDER-2, *R*^2^ = 0.52, AICc = 724.9, *z* = 0.900, *P* = 0.184; Knight ADRC, *R*^2^ = 0.44, AICc = 385.8, *z* = −1.405, *P* = 0.080, Fig. 5). Results for amyloid-positive only participants demonstrated similar results (Supplementary Fig. 4, Supplementary Table 7 and Extended Data Table 4).

## Discussion

In this study, we found that a new CSF biomarker, MTBR-tau243, was strongly associated with tau pathology, whereas it was minimally associated with Aβ pathology, in two large independent sporadic AD cohorts. We also found that CSF MTBR-tau243 has a significantly higher correlation with cognitive measures than phosphorylated tau measures (for example, pT217/T217 and pT181/T181), which indicates its potential utility in the clinical setting. Further, we found that CSF MTBR-tau243 is the biomarker with the largest rate of increase in participants that are already positive for both amyloid and tau pathologies, suggesting that CSF MTBR-tau243 best reflects disease progression in late stages. We further extended these findings by combining CSF MTBR-tau243 with phosphorylated tau measures to predict Aβ pathology, tau pathology and cognitive measures in the AD continuum. We found that CSF MTBR-tau243 in combination with pT205/T205 can accurately predict continuous tau-PET measures and has similar predictive accuracy for cognitive measures as tau-PET. Based on these results, our study suggests that CSF MTBR-tau243 may be a viable alternative to tau-PET for use as a pre-screening tool or a tau pathology end point surrogate for clinical trials and also as an accurate diagnostic measure of tau pathology.

Our first objective was to characterize MTBR-tau243 concentration and compare it to four phosphorylated tau measures by looking at their associations with Aβ and tau pathologies measured by PET. Notably, MTBR-tau243 was the tau biomarker that demonstrated the highest correlation with tau-PET and the lowest correlation with amyloid-PET, not only in the whole group, but also in Aβ-positive group. This suggests that MTBR-tau243 is a biomarker that specifically reflects aggregated tau pathology independent of amyloid pathology. Although pT217/T217 was also well correlated with tau-PET, there was a nonlinear relationship and a substantial increase in pT217/T217 before tau-PET pathology was elevated, which plateaued once the tau-PET threshold was exceeded. This may indicate that pT217/T217 is primarily associated with tau pathology through its quantitative relationship with the amount of Aβ pathology. This is further supported by the observation that Aβ pathology explained a significantly larger proportion of variation of pT217/T217 levels than tau, when including both amyloid-PET and tau-PET measures in the model. Notably, while pT205/T205 levels demonstrated a high correlation with tau-PET, they also showed the second highest correlation with amyloid-PET, after pT217/T217. In combined models, both amyloid-PET and tau-PET explained similar proportion of variation of pT205/T205 levels, suggesting that it is an intermediate biomarker affected by both Aβ and tau pathologies. Regarding the other p-tau measures, pT181/T181 and pT231/T231 were highly correlated with amyloid-PET, while the correlations with tau-PET were significantly lower than the other three CSF tau biomarkers, suggesting that they mainly reflect Aβ-pathology. These results are in line with several recent studies suggesting that p-tau181, p-tau217 and p-tau231 may be more related to amyloid pathology than tau. This is supported by their increased levels, both measured in CSF or in plasma, in early stages^13,14,17,21,22,41–43^, and by being more tightly associated with amyloid-PET than tau-PET^18,23,44^ or to actual amyloid pathology in postmortem studies^20,45^. Finally, we found that MTBR-tau243 was particularity increased in two cases of MAPT R406W mutation carriers that were amyloid negative but had high tau-PET binding. Tau pathology on MAPT R406W mutation carriers is known to be similar to AD tau pathology^46,47^ and reactive to AD tau-PET tracers^4,48–50^, further supporting our finding that MTBR-tau243 is a specific biomarker to AD-like tau pathology.

Longitudinal CSF biomarkers changes were also investigated to understand how these biomarkers change at different stages of the disease. Most notably, among the five CSF tau biomarkers, only MTBR-tau243 exhibited a significant increase in the rate of change between A+T− and A+T+ groups, suggesting that it enables longitudinal disease tracking during the phase of the disease characterized by neocortical tau aggregates, which mainly occurs in the symptomatic phase of AD. On the other hand, there was no major difference in the rate of change between A+T− and A+T+ for pT205/T205, although it still demonstrated a positive rate of changes at this late stage, suggesting a lower but still significant increase after tau deposition. Notably, for the other phosphorylated tau measures (pT181/T181, pT217/T217 and pT231/T231), there was a pronounced increase in the rate of change during the transition from A−T− to A+T−, consistent with a previous report showing that phosphorylated tau (especially p-tau217) is an optimal marker for disease monitoring during the very early (preclinical) stages of the disease^51^. Of note, here we found either no significant increase in the rate of change of phosphorylated tau occupancy during the transition from A+T− to A+T+ or a significant decrease in the rate of change, consistent with previous reports^21^. These results suggest that rate of change in these phosphorylated tau measures may plateau or decline at advanced disease stages, when insoluble tau aggregates are depositing in the neocortex, indicating they are discordant longitudinally and that the classic p-tau measures are not direct measures of AD tau pathology^52^. Altogether, the findings of CSF p-tau measures are consistent with previous clinical observational studies and preclinical mouse models^22,32^, where these biomarkers seem to be driven by Aβ pathology. These results further support recent proposals to revise the A/T/(N) criteria system, in which any p-tau biomarker can be used as a tau (T) marker^53^.

As a relevant question for clinical practice, we also investigated the relationship between these CSF biomarkers and a cognitive measure. As expected by the observed associations with tau pathology, MTBR-tau243 was the measure most strongly associated with MMSE, a cognitive test frequently used in the clinical setting. Notably, this association was not significantly different from tau-PET, thus supporting the idea that CSF MTBR-tau243 could be a viable alternative to tau-PET for clinical purposes. Although pT205/T205 had a lower correlation with MMSE than MTBR-tau243, pT205/T205 was also well correlated with MMSE and not significantly different from tau-PET. In contrast, other CSF biomarkers such as pT217/T217 or pT181/T181 showed significantly lower associations.

An unmet need is to determine not just who has amyloid or tau pathology, but if the symptoms are due to those pathologies. Because tau pathology is most highly correlated with cognitive and clinical impairment, an important question is how well CSF biomarkers can predict tau pathology or cognitive impairment. Thus, we next examined whether combining CSF biomarkers would improve prediction of Aβ or tau pathologies or cognitive measures. Based on a data-driven approach, we observed that the combination of pT205/T205, Aβ42/40 and pT217/T217 was optimal for predicting amyloid-PET continuous measures and significantly improved the performance of any individual measure. We also found that the combination of MTBR-tau243 and pT205/T205 in a single model improved prediction of tau-PET burden compared to any other single-fluid biomarker. The fact that such high predictive accuracy for both amyloid-PET and tau-PET imaging can be achieved by CSF biomarkers indicates that CSF assays can potentially be an alternative to PET measures, which are costly and have limited accessibility. Notably, MTBR-tau243 and pT205/T205 were also the optimal combination for predicting a cognitive measure (MMSE), suggesting potential clinical applications of this biomarker combination in predicting not only tau pathology but also cognitive impairment. For broader use, the translation of these biomarkers into blood-based biomarkers will be of utmost importance.

The main strength of this study is that we replicated our key findings in two large independent cohorts that represented different types of populations, used different PET tracers and also that we measured collected samples prospectively together with predefined outcome measures. Although further research is needed in a more diverse and generalizable population to implement our findings in the clinic, it is important to highlight that BioFINDER-2 participants were consecutively recruited from a secondary Clinical Memory in Sweden. As such, this cohort is a representative of memory clinical patients in Sweden and include both AD and also non-AD dementia patients. Limitations include that the magnitude of the trend differed between the two cohorts in some analyses although similar trends were shown. Potential reasons include that the BioFINDER-2 cohort includes more tau-PET-positive participants with AD dementia than the Knight ADRC cohort, as well as more participants in advanced stages of the disease, which may have affected the results with tau-PET and MMSE. Another limitation is that relatively few participants with AD dementia in the BioFINDER-2 cohort had an amyloid-PET scan per study design, although these participants all had CSF Aβ42/40. Thus, we used CSF Aβ42/40 instead of amyloid-PET as a marker of Aβ pathology in a sensitivity analysis and confirmed that this limitation did not affect the overall results and interpretations. Further, we acknowledge that our measures of Aβ and tau pathologies are only surrogate biomarkers and not actual measures of pathology, but both amyloid-PET and tau-PET markers have been validated against neuropathological measures of insoluble Aβ and tau aggregates, respectively^2,3,6,54–57^. Future studies using animal models and neuropathological measures will be important to further validate the results here presented.

In conclusion, these findings confirm that CSF MTBR-tau243 specifically reflects changes in aggregated tau pathology that occur at a late stage of AD progression and are associated with clinical and cognitive symptoms. Thus, we suggest that MTBR-tau243 should replace the commonly used p-tau measures as the fluid biomarker representing insoluble tau aggregate pathology (T) in defining AD pathology and in future versions of the commonly used A/T/(N) criteria for AD^52^. As such, MTBR-tau243 could be used to assess AD tauopathy and track the effects of drug treatment independent of amyloid effects. The combination of CSF MTBR-tau243 and pT205/T205 is nearly equivalent to tau-PET measures and predicts MMSE almost as accurately as tau-PET, which indicates clinical utility of a biomarker panel containing MTBR-tau243. Compared to biomarkers altered by amyloidosis that are often abnormal in older cognitively normal individuals, CSF MTBR-tau243 could enable confirmation of tau pathology and provide greater certainty that cognitive symptoms are due to AD, as proposed in the latest clinical AD criteria requiring biomarker evidence of both amyloid and tau pathology to diagnose AD with high likelihood^58^. These findings add to the improving biomarker diagnostic accuracy for AD and to strategies to develop new AD therapies.

## Methods

### Participants

Participants were included from two cohorts: the Swedish BioFINDER-2 (NCT03174938) (ref. ^13^) at Lund University (Lund, Sweden) and the Knight ADRC from Washington University. The BioFINDER-2 cohort included cognitively unimpaired participants (recruited as cognitively normal controls or as patients with subjective cognitive decline (SCD)), patients with MCI, patients with AD dementia and patients with a non-AD neurodegenerative disease. Participants were recruited at Skåne University Hospital and the Hospital of Ängelholm in Sweden. Details on recruitment, exclusion and inclusion criteria have been presented before^13^. All participants underwent lumbar puncture at baseline and at the follow-up after 2 years for CSF sampling. Participants underwent cognitive testing, including MMSE. The Knight ADRC cohort consisted of community-dwelling volunteers enrolled in studies of memory and aging at Washington University in St Louis. All Knight ADRC participants underwent a comprehensive clinical assessment that included a detailed interview of a collateral source, a neurological examination of the participant, the Clinical Dementia Rating^®^ (CDR)^59^ and the MMSE^40^. Individuals with a CDR of 0.5 or greater were considered to have a dementia syndrome and the probable etiology of the dementia syndrome was formulated by clinicians based on clinical features in accordance with standard criteria and methods^60^.

In the BioFINDER-2 cohort, participants were divided in CU as either Aβ negative or positive (CU− and CU+, respectively), patients with MCI Aβ positive (MCI+), patients with AD dementia Aβ positive (AD+) or patients with non-AD neurodegeneration, regardless of their Aβ status. Two participants (one in CU− and the other in non-AD) were MAPT R406W mutation carriers with Aβ negative and tau positive. In the Knight ADRC cohort, participants were divided in CU with CDR of 0 either Aβ negative or positive (CU− and CU+, respectively), patients with very mild AD with CDR of 0.5 Aβ positive and patients with AD dementia with CDR ≥ 1 Aβ positive (AD+). In accordance with the research framework by the National Institute on Aging-Alzheimer’s Association study, patients with SCD and cognitively normal controls were considered the CU group^5^. All participants gave written informed consent and ethical approval was granted by the Regional Ethical Committee in Lund, Sweden and the Washington University Human Research Protection Office, respectively.

### Anti-tau antibody generation

Antibodies HJ32.11 and HJ34.8 were generated by immunizing tau knockout mice (The Jackson Laboratory) with either keyhole limpet hemocyanin (KLH) fused to amino acids 225-242 of tau to generate antibody HJ32.11 or to KLH fused to amino acids 226–264 to generate antibody HJ34.8. Spleen cells from immunized mice were fused with P3 hybridoma cells and expanded. Clones were screened by direct ELISA.

### CSF measurements

Measurement of CSF tau species, including p-tau and MTBR-tau243 was performed at Washington University in both cohorts using the newly developed immunoprecipitation/mass spectrometry (IP/MS) method. We developed two new monoclonal antibodies to immune-purify CSF MTBR-tau243 (HJ32.11, which binds near residue 243 and HJ34.8, which binds near residue 260). The procedure of CSF tau analysis is described in Supplementary Fig. 5. The calculation of percent phosphorylation was performed by measuring the phosphorylated peptide and the non-phosphorylated peptide in the same injection and calculating the percent phosphorylation occupancy as % p-tau/t-tau (ref. ^21^).

Additionally, CSF Aβ42/40 levels were used in both cohorts to assess Aβ positivity. In the BioFINDER-2 cohort, CSF levels of Aβ42/40 were measured as previously explained^13^. A threshold of 0.080, based on a Gaussian mixture modeling, determined Aβ positivity^39^. In the Knight ADRC cohort, CSF Aβ42/40 levels were measured as explained previously^61^. The threshold (0.0673) had the maximum combined sensitivity and specificity in distinguishing amyloid-PET status.

### Imaging acquisition and quantification

In the BioFINDER-2 cohort, amyloid and tau-PET acquiring methods have been previously reported^13^. Briefly, amyloid-PET was acquired using [^18^F]flutemetamol and tau-PET using [^18^F]RO948. Of note, most of the patients with AD did not undergo amyloid-PET in BioFINDER-2, due to the study design. In the Knight ADRC cohort, participants underwent amyloid-PET using either [^18^F]florbetapir ([^18^F]AV45) or [^11^C]PiB and tau-PET with [^18^F]flortaucipir ([^18^F]AV1451) as previously explained^33^. Amyloid-PET was measured in a neocortical meta-ROI using cerebellar gray as a reference region. In the BioFINDER-2 cohort, Centiloids were calculated using the Computational Analysis of PET from AIBL (CapAIBL) pipeline^62^. For tau-PET, SUVRs were calculated using the inferior cerebellum cortex as reference region and binding from a temporal meta-ROI were used for main analyses (Braak I–IV), to capture the regions most affected by tau. In supplementary analyses, we also quantified tau-PET in early (Braak I), intermediate (Braak III–IV) and late (Braak V–VI) regions of tau deposition^63^. Tau positivity was assessed based on tau-PET in all cases. In the Braak I–IV region, cutoff for positivity was set at SUVR > 1.32 both in BioFINDER-2 and in the Knight ADRC cohorts^64,65^.

### Cognitive tests

MMSE was used as a measure of global cognition in both cohorts.

### Statistical analyses

Differences in CSF biomarker levels by diagnostic groups were tested using ANCOVA adjusted for age and sex. Post hoc analyses were performed using the Tuckey test. Linear regression models were used to assess the association between amyloid-PET and tau-PET (independent variable) and each of the CSF biomarkers (dependent variable), after adjusting for age and sex. For cognition, we additionally used years of education as covariate in the linear regression models. All standardized β values were compared to the highest for each outcome and cohort, by building a distribution of the β values’ difference and using that to infer significance using a bootstrapping approach (*n* = 500) with the *boot* package. Proportion of variation of CSF levels by amyloid and tau measures were assessed using linear regression models with both amyloid and tau as predictors, CSF levels as outcomes and age and sex as covariates. We calculated the partial *R*^2^ of amyloid and tau, raw and as a percentage of the total *R*^2^ of the model using the *rsq* package. This was used as a measure of proportion of variance explained by amyloid and tau. Next, prediction of amyloid and tau continuous measures was assessed with linear regression models, where amyloid-PET and tau-PET measures were used as outcomes in independent models and individual CSF biomarkers as predictors. A basic model was also created with only covariates (age and sex) as predictors. Additionally, a parsimonious model was constructed to optimally predict (highest accuracy with lower number of predictors) each of these measures, independently for each cohort. To this aim, LASSO regression models were used (*glmnet* package), initially including all CSF biomarkers and covariates. Only those predictors selected by the LASSO regression and with a significant contribution (*P* < 0.1) in the model were finally included in the parsimonious model. Similar methods were used for predicting cognition (MMSE in the two cohorts and CDR in Knight ADRC) additionally including years of education as covariate. In these cases, we compared the parsimonious model to one including only tau-PET as predictor. F-tests were used to compare nested models (including the same subset of predictors). When comparing models with different predictors we used the Vuong’s test using the *nonnest2* package. Finally, CSF longitudinal changes by baseline amyloid and tau status were assessed in the BioFINDER-2 cohort. Individual participant slopes were calculated using linear regression models to calculate rate of change differences (mean percentage change) and compare them between groups using a Kruskal–Wallis test. Further, we created group trajectories with linear mixed models using the *lme4* package for visualization. Here, CSF biomarkers were used as outcome, interaction between time and baseline amyloid and tau status as predictor and age and sex main effects as covariates, using random intercepts and fixed time slopes due to low number of time points. CSF and amyloid-PET and tau-PET measures were log-transformed in linear regression analyses. A two-sided *P* value < 0.05 was considered statistically significant. R v.4.1.0 was used for all statistical analyses.

### Reporting summary

Further information on research design is available in the Nature Portfolio Reporting Summary linked to this article.

## Online content

Any methods, additional references, Nature Portfolio reporting summaries, source data, extended data, supplementary information, acknowledgements, peer review information; details of author contributions and competing interests; and statements of data and code availability are available at 10.1038/s41591-023-02443-z.

### Supplementary information

Supplementary Information Supplementary Figs. 1–5 and Supplementary Tables 1–7.

Reporting summary

## Figures and Tables

**Extended Data Table 1:**
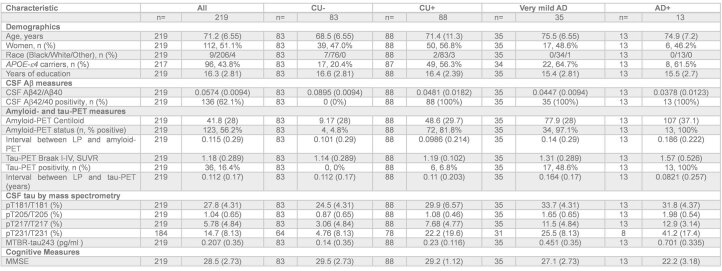
Knight ADRC participants characteristics

Abbreviations: AD + , Alzheimer’s disease dementia amyloid positive; CSF, cerebrospinal fluid; CU-, cognitively unimpaired amyloid negative; CU + , cognitively unimpaired amyloid positive; MMSE, Mini-Mental State Examination; MTBR, microtubule binding region; PET, positron emission tomography; SUVR, standardized uptake value ratio.

Parenthesis in rows: standard deviation.

**Extended Data Table 2:**
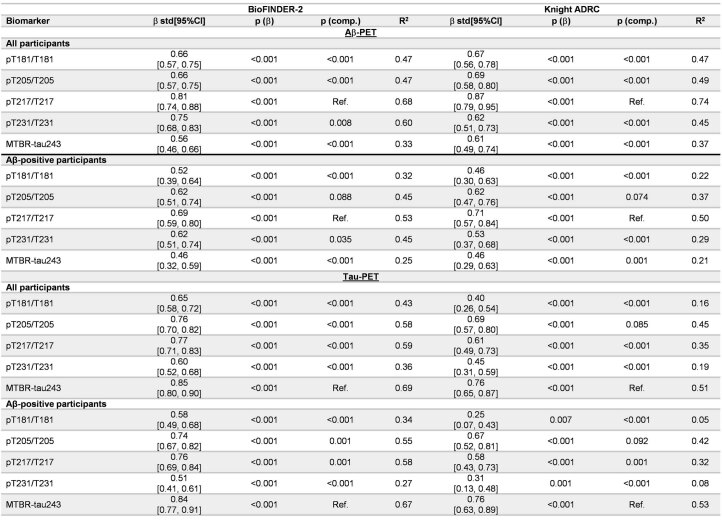
Associations between CSF biomarkers and Aβ-PET and tau-PET

Linear regression models were used to assess the associations between CSF biomarkers and amyloid-PET (Centiloids) and tau-PET (SUVR) in Braak I-IV ROI adjusting for age and sex. Amyloid-positive participants were selected based on CSF Aβ42/40 previously validated cutoffs (CSF Aβ42/40 < 0.08 in BioFINDER-2 and CSF Aβ42/40 < 0.0673 in Knight ADRC). P comparison (p comp.) was calculated to assess differences between the strongest association (Ref.) and each of the other CSF biomarkers using bootstrapping (n = 500) from adjusted β. Significant p comparison ( < 0.05) suggests weaker associations.

Abbreviations: Aβ, amyloid; CI, confidence interval; CSF, cerebrospinal fluid; MTBR, microtubule binding region; PET, positron emission tomography; SUVR, standardized uptake value ratio.

**Extended Data Table 3:**
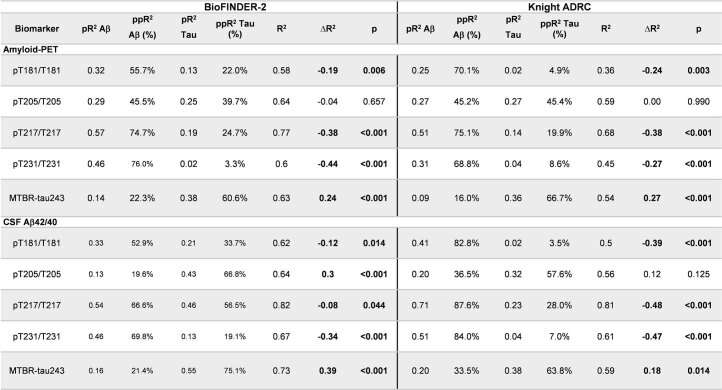
Proportion of variation of CSF biomarker levels explained by amyloid and tau

Proportion of variation of CSF biomarker levels explained by amyloid and tau was calculated by partial R^2^ (pR^2^) in multivariable linear regression models with amyloid (CSF Aβ42/40 [top rows] or amyloid-PET [bottom rows]) and tau (tau-PET in Braak I-IV ROI) as predictors and each CSF biomarker as outcome, adjusting for age and sex. Percentual partial R^2^ (ppR^2^) were calculated as the partial R^2^ of each predictor divided by the total R^2^ of the model (100 * pR^2^/R^2^). ΔR^2^ represents the difference between amyloid and tau partial R^2^, with positive values meaning more proportion of variance explained by tau (ΔR^2^ tau - ΔR^2^ Aβ). Differences on partial R^2^ were assessed by bootstrapping, and significant p-values (p < 0.05, in bold) represent significant differences on the contribution of tau and amyloid pathologies on each of the CSF levels.

Abbreviations: Aβ, amyloid; CSF, cerebrospinal fluid; MTBR, microtubule binding region.

**Extended Data Table 4:**
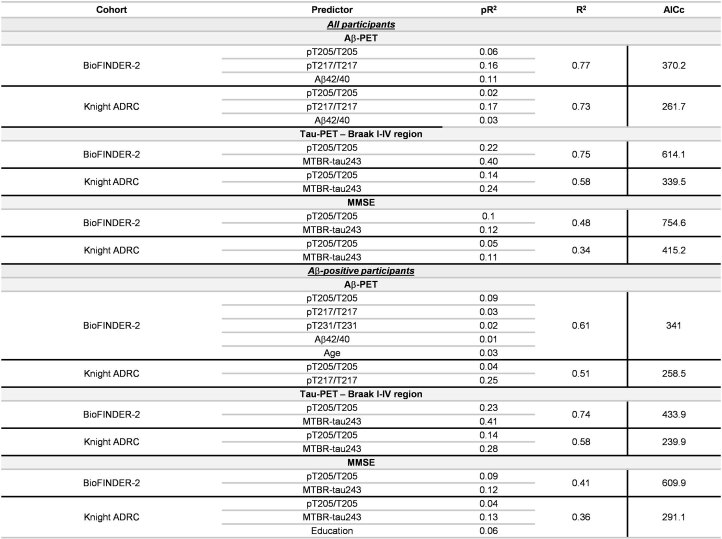
Parsimonious models for predicting AD-related continuous measures

Partial R^2^ (pR^2^) for all predictors included in the parsimonious model were calculated in a multivariable linear regression model with amyloid-PET, tau-PET and MMSE continuous measures as outcomes, respectively. R^2^ and AICc for the whole model are also included in the table. Parsimonious model for each outcome and cohort were obtained with the optimal combination of CSF biomarkers and demographics (age and/or sex and/or education) assessed using a LASSO regression. Non-AD cases were excluded from BioFINDER-2 cohort for the MMSE analyses. Amyloid-positive participants were selected based on CSF Aβ42/40 previously validated cutoffs (CSF Aβ42/40 < 0.08 in BioFINDER-2 and CSF Aβ42/40 < 0.0673 in Knight ADRC).

Abbreviations: Aβ, amyloid; AICc, corrected Akaike information criterion; CSF, cerebrospinal fluid; LASSO, least absolute shrinkage and selection operator; MMSE, Mini-Mental State Examination; MTBR, microtubule binding region; PET, positron emission tomography.

## Data Availability

The datasets generated and/or analyzed during the current study are available from the corresponding authors (R.J.B. and O.H.). We will share datasets within the restrictions of institutional review board ethics approvals, upon reasonable request. Pseudonymized data from the BioFINDER-2 will be shared by request from a qualified academic investigator for the sole purpose of replicating procedures and results presented in the article and as long as data transfer is in agreement with EU legislation on the General Data Protection Regulation and decisions by the Ethical Review Board of Sweden and Region Skåne, which should be regulated in a material transfer agreement. Knight ADRC data are available to qualified investigators who have a proposal approved by an institutional committee (https://knightadrc.wustl.edu/Research/ResourceRequest.htm) that meets monthly. The study must be approved by an institutional review board to ensure ethical research practices and investigators must agree to the terms and conditions of the data use agreement, which includes not distributing the data without permission.
