# Supplementary material for: CSF MTBR-tau243 is a specific biomarker of tau tangle pathology in Alzheimer’s disease
Source: Nat Med. 2023 Jul 13;29(8):1954–63. doi: 10.1038/s41591-023-02443-z (PMC10427417; doi:10.1038/s41591-023-02443-z)
Supplement: Supplementary file 1 — Supplementary Figs. 1–5 and Supplementary Tables 1–7. [file 41591_2023_2443_MOESM1_ESM.pdf]

nature medicine

Article

https://doi.org/10.1038/s41591-023-02443-z

CSF MTBR-tau243 is a specific biomarker of tau tangle pathology in Alzheimer's disease

In the format provided by the authors and unedited

Supplementary Fig. 1: **Associations between all CSF biomarkers and CSF Aβ42/40**

Supplementary Fig. 2: **Proportion of variation of CSF biomarker levels explained by CSF Aβ42/40 and tau-PET**

Supplementary Fig. 3: **Longitudinal CSF biomarkers change by baseline amyloid and tau status**

Supplementary Fig. 4: **Predicting AD-related continuous measure by CSF biomarkers in amyloid positive participants**

Supplementary Fig. 5: **Method of CSF tau analysis**

Supplementary Table 1: **CSF biomarkers by diagnosis**

Supplementary Table 2: **Associations between CSF biomarkers and CSF Aβ42/40**

Supplementary Table 3: **Associations between CSF biomarkers and tau-PET SUVR in different Braak regions**

Supplementary Table 4: **Characteristics of BioFINDER-2 participants with longitudinal CSF available**

Supplementary Table 5: **Longitudinal CSF biomarkers change by baseline AT status**

Supplementary Table 6: **Associations between AD-biomarkers and MMSE**

Supplementary Table 7: **Predicting AD-related continuous measure by CSF biomarkers**

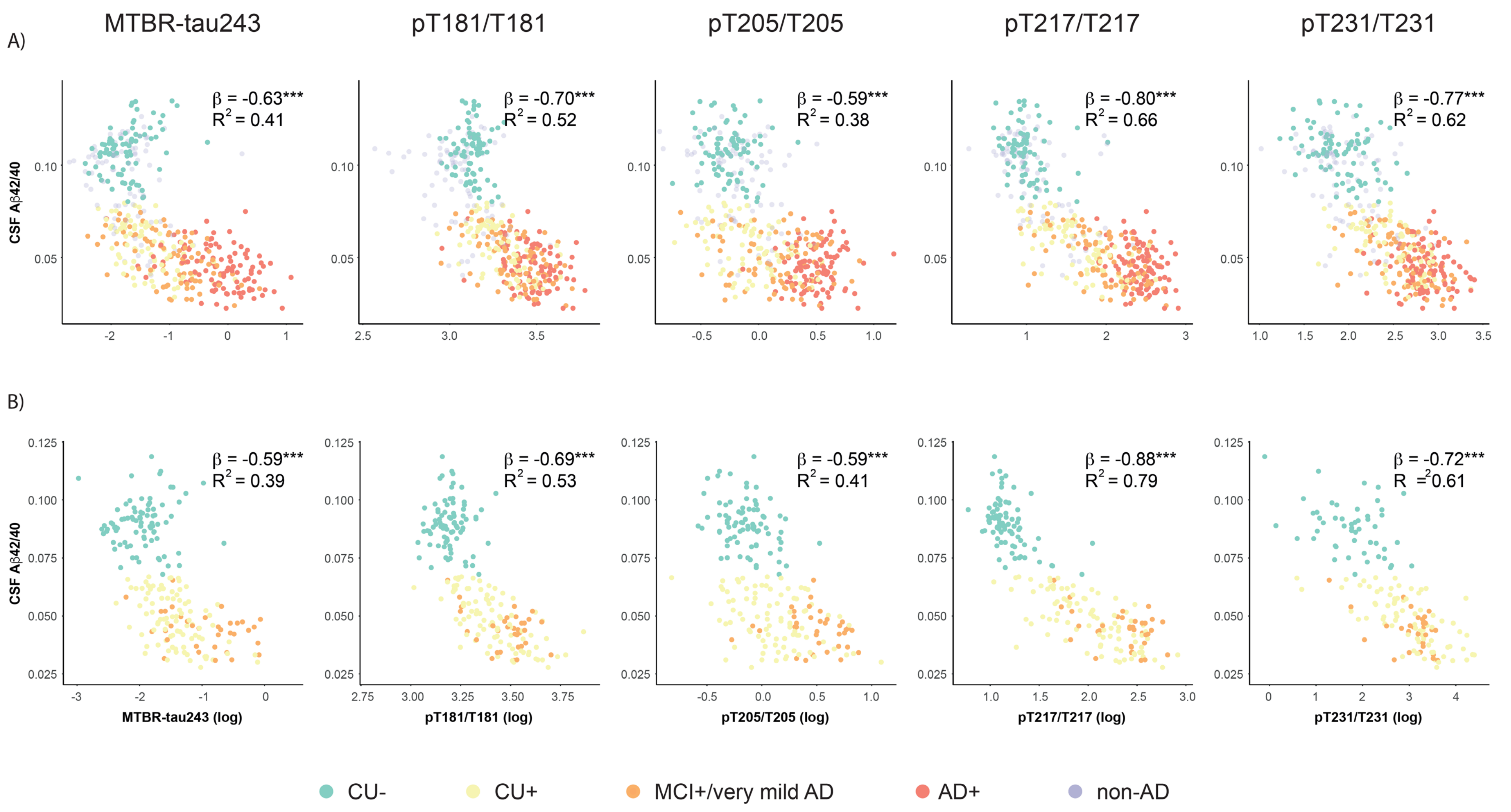

Supplementary Fig. 1: Associations between all CSF biomarkers and CSF Aβ42/40

Associations between CSF biomarkers and CSF Aβ42/40 in BioFINDER-2 (A, n=427) and Knight ADRC (B, n=219; except for pT231/T231 in which n=184) participants. Linear regression models, adjusting for age and sex, were used to obtain standardized β, p-values (asterisks) and R^2 are shown in the plots. P-values were based on two-sided tests unadjusted for multiple comparisons. *, p<0.050; **, p<0.010; ***, p<0.001.

Abbreviations: AD+, Alzheimer's disease dementia amyloid positive; CL, Centiloids, CSF, cerebrospinal fluid; CU-, cognitively unimpaired amyloid negative; CU+, cognitively unimpaired amyloid positive; MCI+, mild cognitive impairment amyloid positive; MTBR, microtubule binding region; non-

AD, non-Alzheimer's disease dementia; PET, positron emission tomography.

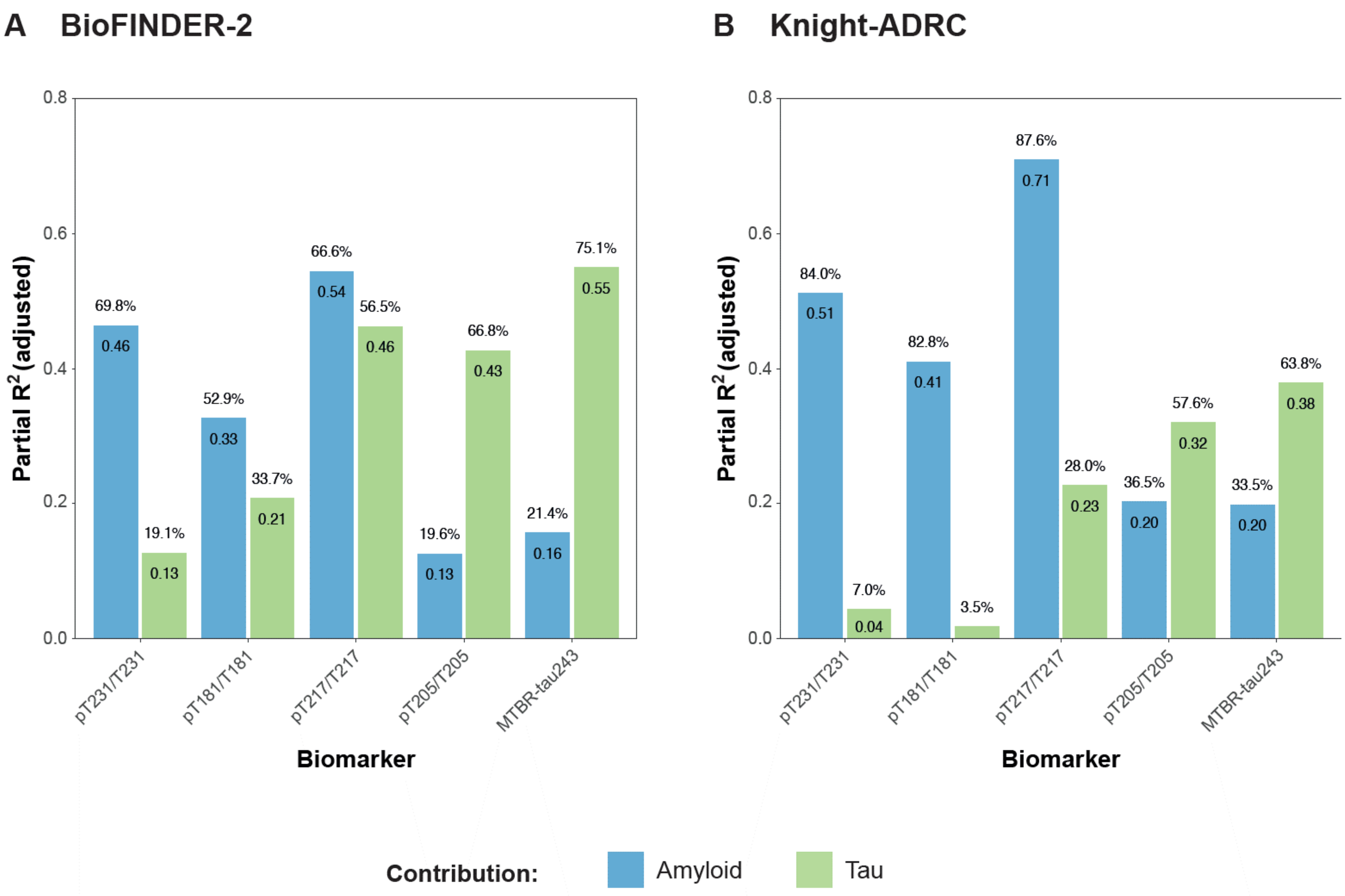

Supplementary Fig. 2: Proportion of variation of CSF biomarker levels explained by CSF Aβ42/40 and tau-PET

Proportion of variation of CSF biomarker levels explained by CSF Aβ42/40 and tau-PET is shown as partial R^2 for each biomarker in BioFINDER-2

(A, n=422) and Knight ADRC (B, n=184). Partial R^2 values are shown inside columns. Percentages of partial R^2 over the total R^2 of the model are shown on top of each column. Partial R^2 and total R^2 were computed using each CSF biomarker as outcome and amyloid and tau measures as predictors in a linear regression model adjusted for age and sex, in independent models for each CSF biomarker and cohort. Percentages do not add to 100% as possible shared variance. Biomarkers are ordered (from left to right) by the increasing contribution (%) of tau on their levels.

Abbreviations: CSF, cerebrospinal fluid; CU-, cognitively unimpaired amyloid negative; MTBR, microtubule binding region; PET, positron emission tomography.

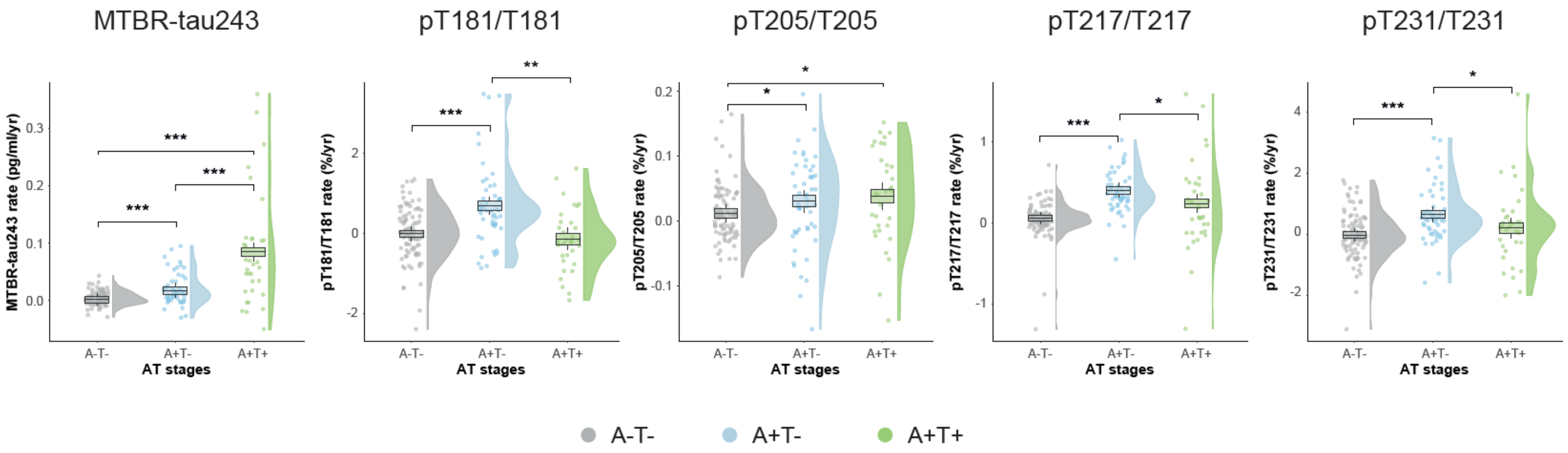

Supplementary Fig. 3: Longitudinal CSF biomarkers change by baseline amyloid and tau status

CSF biomarker rates of change per baseline amyloid (A), as measured by PET, and tau (T), as measured by PET, status (n=174). Dots represent individual rates of change. Boxplots represent trajectories per group as assessed by linear mixed models (central band of the boxplot represents the median of the group, the lower and upper hinges correspond to the first and third quartiles, and the whiskers represent the maximum/minimum value or the 1.5 IQR from the hinge, whatever is lower). Differences among all groups were tested using a Kruskal-Wallis tests and pairwise Wilcoxon tests for post-hoc comparisons. P-values come from two-sided tests uncorrected for multiple comparisons and are represented by asterisks. Longitudinal CSF data was only available in BioFINDER-2. Amyloid-positive participants were selected based on amyloid-PET previously validated cut-offs (SUVR>1.03). Tau positivity was assessed based on tau-PET SUVR in the meta-ROI (SUVR>1.32). Actual p-values for A-T- vs. A+T- were: p=0.024 (pT205/T205); for A-T- vs. A+T+ were: p=0.277 (pT181/T181), p=0.041 (pT205/T205), p=0.178 (pT217/T217) and p=0.368 (pT231/T231); and for A+T- vs. A+T+ were: p=0.001 (pT181/T181), p=0.738 (pT205/T205), p=0.011 (pT217/T217), p=0.023 (pT231/T231). The rest of comparisons rendered a p<0.001.*, p<0.050; **, p<0.010; ***, p<0.001.

Abbreviations: A-T-, amyloid and tau negative; A+T-, amyloid positive and tau negative; A+T+, amyloid positive, tau positive; CSF, cerebrospinal fluid; MTBR, microtubule binding region; PET, positron emission tomography; SUVR, standardized uptake value ratio.

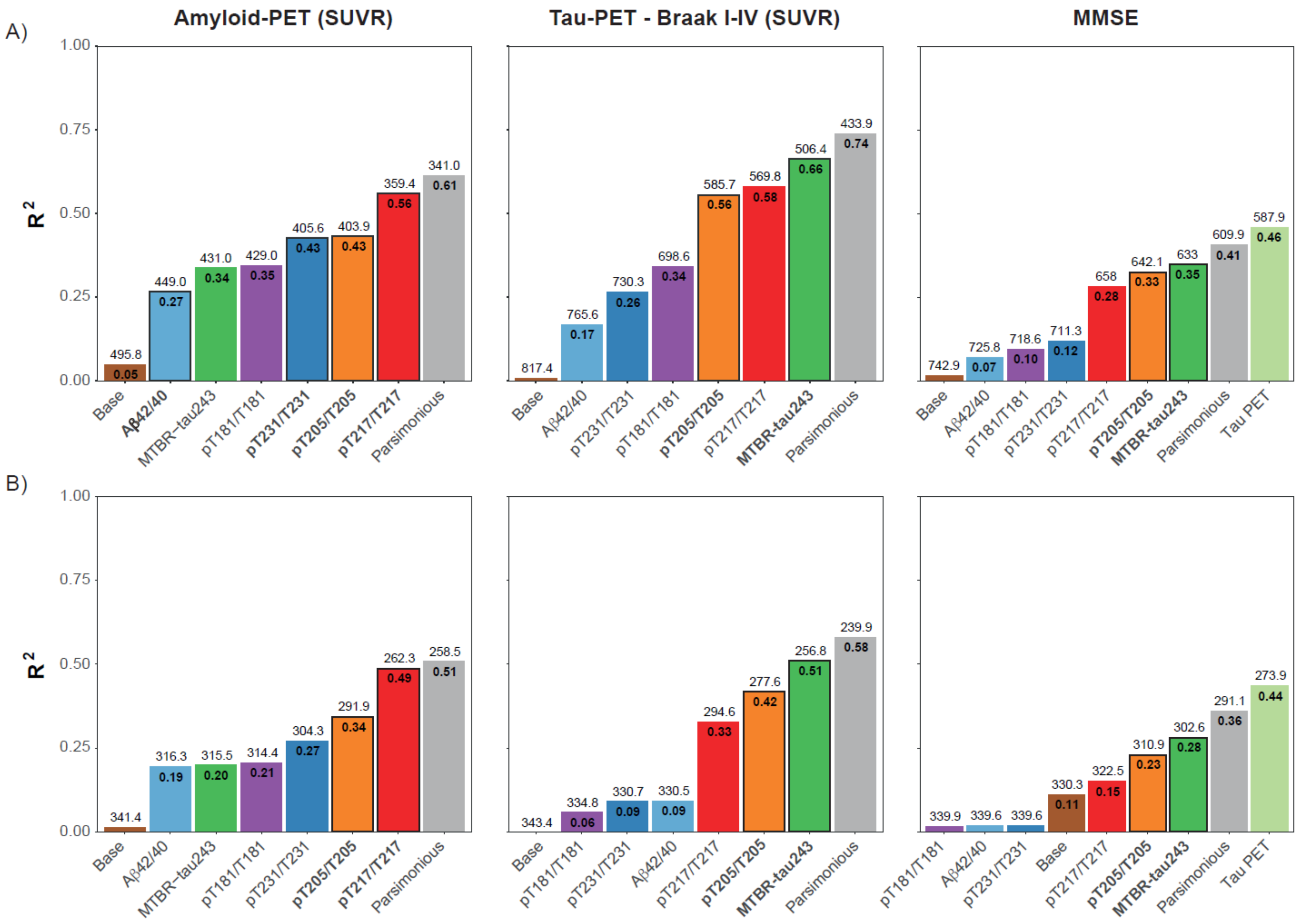

Supplementary Fig. 4: Predicting AD-related continuous measure by CSF biomarkers in amyloid-positive participants

Linear regression models were used for predicting amyloid-PET (first column, n=172), tau-PET (second column, n=287) and MMSE (third column, n=261) in BioFINDER-2 (A) and Knight ADRC (B, n=117) amyloid positive participants. Base model included age and sex (and education for MMSE) as predictors. Parsimonious model was obtained with the optimal combination of CSF biomarkers and demographics (age and/or sex and/or education) assessed using a LASSO regression. Biomarkers included in the parsimonious models are depicted a black border and their name is shown in bold. The other models used only individual CSF biomarkers as predictors. CSF Aβ42/40 and tau-PET were used as predictor in independent models for predicting cognition as a comparison. Models were compared using an F-test (nested models) or Vuong's test (non-nested models). Non-AD cases were excluded from BioFINDER-2 cohort for the cognition analyses. Amyloid-positive participants were selected based on CSF Aβ42/40 previously validated cut-offs (CSF Aβ42/40<0.08 in BioFINDER-2 and CSF Aβ42/40<0.0673 in Knight ADRC).

Abbreviations: Aβ, amyloid-β; CSF, cerebrospinal fluid; CU-, cognitively unimpaired amyloid negative; MMSE, Mini-Mental State Examination; LASSO, least absolute shrinkage and selection operator; MTBR, microtubule binding region; PET, positron emission tomography.

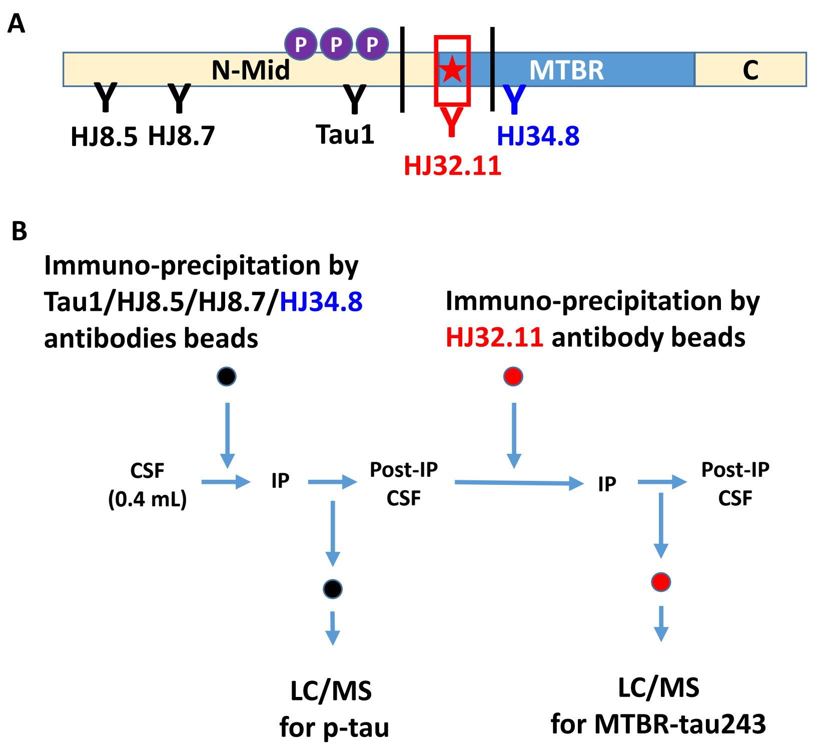

Supplementary Fig. 5: Method of CSF tau analysis

(A) Schematic of binding sites of antibodies used in this study and (B) the sample preparation scheme. CSF tau species were immunoprecipitated by Tau1 (generated by Dr. Nicholas Kanaan) and HJ series (clone name: HJ8.5, HJ8.7, HJ32.11, and HJ34.8) antibodies (generated by Dr. David Holtzman). For all antibodies, 3 mg/gram sepharose beads were generated. For Tau1 immunoprecipitation (IP), 1.1 ug antibody/sample was used. For HJ8.5 and HJ8.7 IP, 2.3 ug antibody/sample was used. For HJ32.11 and HJ34.8 IP, 11.25 ug antibody/sample was used. CSF (450 µL) was mixed with 20 µL of solution containing 100 pg/µL each of ^{15}N 0N3R-tau and ^{15}N 2N4R-tau as internal standards. The tau species consisting primarily of N-terminal to mid-domain regions and late MTBR region (around the residue 260 in R1) were immunoprecipitated with Tau1, HJ8.5, HJ8.7, and HJ34.8 antibodies. Subsequently, 20 µL of $^{13}C^{15}N$ 2N4R-tau internal standard (100 pg/µL) was spiked into the post-immunoprecipitated CSF. Then, the MTBR-tau243 species containing the residue 243 was immunoprecipitated with the other novel antibody HJ32.11 in the post-immunoprecipitated CSF. All immunoprecipitated tau species (p-tau and MTBR-tau243) were digested by trypsin and desaltated by C18 toptip (Glygen)[9,33]. The resulting tau peptides were analyzed by nanoAcquity ultra-performance LC system (Waters) coupled to Orbitrap Tribrid Eclipse mass spectrometer (Thermo Scientific) operating in parallel reaction monitoring mode. MS transitions were extracted using Skyline v.22.2.2.278 (MacCoss lab, University of Washington). Data were aggregated using Tableau v.2022.2.2 (Tableau Software) to calculate CSF biomarker levels.

Biomarker	pT181/T181	pT205/T205	p217/T217	pT231/T231	MTBR-tau243
**CU+ vs. CU-**	<0.001	0.002	<0.001	<0.001	0.466
**MCI+ vs. CU-**	<0.001	<0.001	<0.001	<0.001	<0.001
**ADD+ vs. CU-**	<0.001	<0.001	<0.001	<0.001	<0.001
**PSP/CBS vs. CU-**	0.560	0.431	0.354	0.402	1
**FTD/PPA vs. CU-**	0.440	0.988	0.918	0.999	0.980
**PD/PDD vs. CU-**	1	0.964	0.960	0.737	1
**MCI+ vs. CU+**	0.001	<0.001	<0.001	0.015	0.003
**ADD+ vs. CU+**	<0.001	<0.001	<0.001	<0.001	<0.001
**PSP/CBS vs. CU+**	<0.001	0.968	0.003	<0.001	0.860
**FTD/PPA vs. CU+**	<0.001	0.25	<0.001	<0.001	0.995
**PD/PDD vs. CU+**	<0.001	0.428	<0.001	<0.001	0.668
**ADD+ vs. MCI+**	<0.001	<0.001	<0.001	<0.001	<0.001
**PSP/CBS vs. MCI+**	<0.001	<0.001	<0.001	<0.001	0.001
**FTD/PPA vs. MCI+**	<0.001	<0.001	<0.001	<0.001	0.006
**PD/PDD vs. MCI+**	<0.001	<0.001	<0.001	<0.001	<0.001
**PSP/CBS vs. ADD+**	<0.001	<0.001	<0.001	<0.001	<0.001
**FTD/PPA vs. ADD+**	<0.001	<0.001	<0.001	<0.001	<0.001
**PD/PDD vs. ADD+**	<0.001	<0.001	<0.001	<0.001	<0.001
**FTD/PPA vs. PSP/CBS**	1	0.935	0.974	0.825	0.998
**PD/PDD vs. PSP/CBS**	0.780	0.977	0.963	0.999	1
**PD/PDD vs. FTD/PPA**	0.706	1	1	0.971	0.979

Supplementary Table 1: CSF biomarkers by diagnosis

Actual p-values of the differences in CSF biomarker levels (BioFINDER-2, n=448) by diagnostic groups were tested using ANCOVA adjusted for age and sex. Post-hoc analyses were performed two-sided using the Tuckey test. Amyloid-positive participants were selected based on CSF Aβ42/40 (CSF Aβ42/40<0.08).

Abbreviations: AD+, Alzheimer's disease dementia amyloid positive; CBS, corticobasal syndrome; CU-, cognitively unimpaired amyloid negative; CU+, cognitively unimpaired amyloid positive; FTD, frontotemporal dementia; MCI+, mild cognitive impairment amyloid positive; MTBR, microtubule binding region; PD, Parkinson's disease; PDD, Parkinson's disease dementia; PPA, primary progressive aphasia; PSP, progressive supranuclear palsy.

	BioFINDER-2				Knight ADRC			
**Biomarker**	**β std [95%CI]**	**p (β)**	**p (comp.)**	**R^2**	**β std [95%CI]**	**p (β)**	**p (comp.)**	**R^2**
**All participants**								
pT181/T181	-0.70 [-0.77, -0.63]	<0.001	<0.001	0.52	-0.69 [-0.79, -0.58]	<0.001	<0.001	0.53
pT205/T205	-0.59 [-0.67, -0.52]	<0.001	<0.001	0.38	-0.59 [-0.71, -0.48]	<0.001	<0.001	0.41
pT217/T217	-0.8 [-0.86, -0.74]	<0.001	Ref.	0.66	-0.88 [-0.95, -0.81]	<0.001	Ref.	0.79
pT231/T231	-0.77 [-0.83, -0.71]	<0.001	0.026	0.62	-0.72 [-0.81, -0.63]	<0.001	0.001	0.61
MTBR-tau243	-0.63 [-0.70, -0.55]	<0.001	<0.001	0.41	-0.59 [-0.71, -0.47]	<0.001	<0.001	0.39

Supplementary Table 2: Associations between CSF biomarkers and CSF Aβ42/40

Linear regression models were used to assess the associations between CSF biomarkers and CSF Aβ42/40 adjusting for age and sex (BioFINDER-2: n=427, Knight ADRC: n=219; except for pT231/T231 in which n=184). P comparison (p comp.) was calculated to assess differences between the strongest association (Ref.) and each of the other CSF biomarkers using bootstrapping (n=500) from adjusted β. Significant p comparison (<0.05) suggests weaker associations. Association p-values were based on two-sided tests and bootstrapping p-values from one-sided tests, all unadjusted for multiple comparisons.

Abbreviations: Aβ, amyloid; CI, confidence interval; CSF, cerebrospinal fluid; MTBR, microtubule binding region.

	All participants								Amyloid-positive participants							
	BioFINDER-2				Knight ADRC				BioFINDER-2				Knight ADRC			
**Biomarker**	**β std [95%CI]**	**p (β)**	**p (comp.)**	**R^2**	**β std [95%CI]**	**p (β)**	**p (comp.)**	**R^2**	**β std [95%CI]**	**p (β)**	**p (comp.)**	**R^2**	**β std [95%CI]**	**p (β)**	**p (comp.)**	**R^2**
**Braak I-II**																
pT181/T181	0.68 [0.61, 0.75]	<0.001	<0.001	0.48	0.43 [0.30, 0.56]	<0.001	<0.001	0.23	0.63 [0.53, 0.72]	<0.001	<0.001	0.40	0.26 [0.08, 0.44]	0.005	<0.001	0.07
pT205/T205	0.77 [0.71, 0.83]	<0.001	0.001	0.60	0.62 [0.50, 0.74]	<0.001	0.474	0.40	0.76 [0.69, 0.84]	<0.001	0.018	0.58	0.58 [0.43, 0.74]	<0.001	0.887	0.33
pT217/T217	0.81 [0.75, 0.86]	<0.001	0.007	0.65	0.58 [0.46, 0.70]	<0.001	0.119	0.36	0.78 [0.71, 0.86]	<0.001	0.039	0.62	0.49 [0.33, 0.65]	<0.001	0.093	0.24
pT231/T231	0.64 [0.57, 0.71]	<0.001	<0.001	0.42	0.43 [0.29, 0.57]	<0.001	<0.001	0.21	0.54 [0.44, 0.64]	<0.001	<0.001	0.30	0.25 [0.08, 0.43]	0.005	<0.001	0.07
MTBR-tau243	0.85 [0.80, 0.91]	<0.001	Ref.	0.70	0.65 [0.53, 0.77]	<0.001	Ref.	0.42	0.83 [0.76, 0.9]	<0.001	Ref.	0.66	0.59 [0.44, 0.75]	<0.001	Ref.	0.34
**Braak III-IV**																
pT181/T181	0.65 [0.57, 0.72]	<0.001	<0.001	0.42	0.28 [0.13, 0.42]	<0.001	<0.001	0.09	0.57 [0.48, 0.67]	<0.001	<0.001	0.33	0.19 [0.01, 0.37]	0.043	<0.001	0.01
pT205/T205	0.76 [0.70, 0.82]	<0.001	<0.001	0.57	0.56 [0.43, 0.69]	<0.001	0.470	0.30	0.74 [0.66, 0.82]	<0.001	0.001	0.55	0.60 [0.45, 0.76]	<0.001	0.47	0.33
pT217/T217	0.77	<0.001	<0.001	0.58	0.44	<0.001	0.001	0.19	0.76	<0.001	0.001	0.57	0.45	<0.001	0.004	0.18

	[0.70, 0.83]				[0.30, 0.58]				[0.68, 0.83]				[0.28, 0.62]			
pT231/T231	0.59 [0.52, 0.67]	<0.001	<0.001	0.36	0.32 [0.17, 0.47]	<0.001	<0.001	0.11	0.51 [0.41, 0.61]	<0.001	<0.001	0.26	0.20 [0.01, 0.38]	0.034	<0.001	0.02
MTBR-tau243	0.84 [0.79, 0.90]	<0.001	Ref.	0.68	0.59 [0.46, 0.72]	<0.001	Ref.	0.32	0.84 [0.77, 0.90]	<0.001	Ref.	0.67	0.64 [0.49, 0.79]	<0.001	Ref.	0.37
**Braak V-VI**																
pT181/T181	0.57 [0.49, 0.65]	<0.001	<0.001	0.32	0.36 [0.21, 0.50]	<0.001	<0.001	0.13	0.52 [0.42, 0.62]	<0.001	<0.001	0.28	0.21 [0.03, 0.40]	0.022	<0.001	0.02
pT205/T205	0.69 [0.62, 0.76]	<0.001	0.013	0.46	0.65 [0.54, 0.77]	<0.001	0.095	0.40	0.68 [0.60, 0.77]	<0.001	0.009	0.47	0.64 [0.49, 0.79]	<0.001	0.085	0.37
pT217/T217	0.67 [0.60, 0.75]	<0.001	<0.001	0.44	0.56 [0.44, 0.69]	<0.001	<0.001	0.30	0.68 [0.60, 0.77]	<0.001	<0.001	0.47	0.53 [0.37, 0.69]	<0.001	<0.001	0.26
pT231/T231	0.52 [0.44, 0.6]	<0.001	<0.001	0.27	0.40 [0.25, 0.54]	<0.001	<0.001	0.15	0.47 [0.37, 0.57]	<0.001	<0.001	0.23	0.26 [0.09, 0.44]	0.004	<0.001	0.05
MTBR-tau243	0.76 [0.69, 0.82]	<0.001	Ref.	0.54	0.73 [0.61, 0.84]	<0.001	Ref.	0.47	0.76 [0.68, 0.84]	<0.001	Ref.	0.56	0.73 [0.59, 0.86]	<0.001	Ref.	0.47

Supplementary Table 3: Associations between CSF biomarkers and tau-PET SUVR in different Braak regions

Linear regression models were used to assess the associations between CSF biomarkers and tau-PET in different Braak regions (SUVR) adjusting for age and sex (BioFINDER-2: n=443, Knight ADRC: n=219; except for pT231/T231 in which n=184). Amyloid-positive participants (BioFINDER-2:

n=287, Knight ADRC: n=136; except for pT231/T231 in which n=117) were selected based on CSF Aβ42/40 previously validated cut-offs (CSF Aβ42/40<0.08 in BioFINDER-2 and CSF Aβ42/40<0.0673 in Knight ADRC). P comparison (p comp.) was calculated to assess differences between the strongest association (Ref.) and each of the other CSF biomarkers using bootstrapping (n=500) from adjusted β. Significant p comparison (<0.05) suggests weaker associations. Association p-values were based on two-sided tests and bootstrapping p-values from one-sided tests, all unadjusted for multiple comparisons.

Abbreviations: Aβ, amyloid; CI, confidence interval; CSF, cerebrospinal fluid; MTBR, microtubule binding region; PET, positron emission tomography.

	Overall		CU-		CU+		MCI+		AD +		Non-AD	
	n=	220	n=	79	n=	49	n=	44	n=	29	n=	19
**Demographics**												
Age, years	220	73.0 (8.74)	79	72.0 (9.48)	49	74.3 (7.72)	44	73.1 (9.27)	29	75.7 (7.73)	19	69.9 (7.25)
Women, n (%)	220	107 (48.6%)	79	39 (49.4%)	49	23 (46.9%)	44	23 (52.3%)	29	16 (55.2%)	19	6 (31.6%)
APOE-e4 carriers, n (%)	220	121 (55.0%)	79	26 (32.9%)	49	36 (73.5%)	44	33 (75.0%)	29	21 (72.4%)	19	5 (26.3%)
Years of education	220	12.1 (3.94)	79	11.9 (3.21)	49	12.0 (3.52)	44	12.8 (5.21)	29	11.4 (3.83)	19	13.0 (4.55)
**Amyloid- and tau-status**												
AT status, n (%)	220		79		49		44		29		19	
A-T-		93 (42.3%)		79 (100%)		0 (0%)		0 (0%)		0 (0%)		14 (73.7%)
A+T-		66 (30.0%)		0 (0%)		40 (81.6%)		21 (47.7%)		0 (0%)		5 (26.3%)
A+T+		61 (27.7%)		0 (0%)		9 (18.4%)		23 (52.3%)		29 (100%)		0 (0%)
**CSF tau by mass spectrometry**												
pT181/T181 (%)	220	0.09 (1.00)	79	-0.05 (0.73)	49	0.42 (1.09)	44	0.24 (0.79)	29	-0.36 (1.61)	19	0.17 (0.62)
pT205/T205 (%)	220	0.021 (0.064)	79	0.008 (0.043)	49	0.024 (0.058)	44	0.043 (0.074)	29	0.026 (0.096)	19	0.015 (0.059)
pT217/T217 (%)	220	0.15 (0.41)	79	0.02 (0.22)	49	0.30 (0.29)	44	0.35 (0.47)	29	-0.01 (0.70)	19	0.10 (0.17)
pT231/T231 (%)	218	0.18 (1.10)	79	-0.02 (0.79)	49	0.38 (0.92)	44	0.50 (1.19)	28	-0.04 (1.82)	18	0.03 (0.77)
MTBR-tau243 (pg/ml)	220	0.035 (0.073)	79	0.003 (0.012)	49	0.017 (0.026)	44	0.067 (0.091)	29	0.125 (0.116)	19	0.002 (0.008)

Supplementary Table 4: Characteristics of BioFINDER-2 participants with longitudinal CSF available

Longitudinal CSF data was only available in BioFINDER-2. Amyloid-positive participants were selected based on CSF Aβ42/40 previously validated cut-offs (CSF Aβ42/40<0.08 in BioFINDER-2). Tau positivity was assessed based on tau-PET SUVR in the meta-ROI (SUVR>1.32 in both BioFINDER-2). Amyloid (A) and tau (T) status are shown in table.

Abbreviations: AD+, Alzheimer's disease dementia amyloid positive; CSF, cerebrospinal fluid; CU-, cognitively unimpaired amyloid negative; CU+, cognitively unimpaired amyloid positive; MCI+, mild cognitive impairment amyloid positive; MTBR, microtubule binding region; non-AD, non-Alzheimer's disease dementia. Parenthesis in rows: standard deviation

	A-T- vs A+T-		A-T- vs A+T+		A+T- vs A+T+	
**Biomarker**	Cohen's d	p	Cohen's d	p	Cohen's d	p
**CSF Aβ42/40**						
**pT181/T181**	0.66	<0.001	-0.22	0.011	-0.69	<0.001
**pT205/T205**	0.38	0.011	0.44	0.014	0.08	0.788
**pT217/T217**	**1.39**	<0.001	0.21	0.617	-0.56	<0.001
**pT231/T231**	0.71	<0.001	0.02	0.98	-0.48	0.007
**MTBR-tau243**	0.78	<0.001	**1.48**	<0.001	**1.13**	<0.001
**Amyloid-PET**						
**pT181/T181**	0.78	<0.001	-0.17	0.277	-0.8	0.001
**pT205/T205**	0.32	0.024	0.42	0.041	0.06	0.738
**pT217/T217**	**1.34**	<0.001	0.42	0.178	-0.54	0.011
**pT231/T231**	0.82	<0.001	0.14	0.368	-0.58	0.023
**MTBR-tau243**	0.78	<0.001	**1.72**	<0.001	**1.12**	<0.001

Supplementary Table 5: Longitudinal CSF biomarkers change by baseline AT status

Differences in CSF longitudinal rates of change by baseline Amyloid (A) and Tau (T) status was assessed using were tested using a Kruskal-Wallis tests and pairwise Wilcoxon tests for *post-hoc* comparisons. P-values come from two-sided tests uncorrected for multiple comparisons. Cohen's d among different groups were calculated from individual participant slopes from linear regression models. Amyloid-positive participants were selected based on CSF Aβ42/40 previously validated cut-offs (CSF Aβ42/40<0.08 in BioFINDER-2, n=220, except for pT231/T231 in which n=218) or amyloid-PET (SUVR>1.03, n=174). Tau positivity was assessed based on tau-PET SUVR in the Braak I-IV ROI (SUVR>1.32). The greatest rate of change per A/T group is shown in bold.

	BioFINDER-2				Knight ADRC			
**Biomarker**	**β std[95%CI]**	**p (β)**	**p (comp.)**	**R^2**	**β std[95%CI]**	**p (β)**	**p (comp.)**	**R^2**
**All participants**								
pT181/T181	-0.44 [-0.54, -0.35]	<0.001	<0.001	0.21	-0.24 [-0.38, -0.10]	0.001	<0.001	0.15
pT205/T205	-0.63 [-0.72, -0.55]	<0.001	0.328	0.40	-0.48 [-0.6, -0.35]	<0.001	0.262	0.30
pT217/T217	-0.6 [-0.69, -0.52]	<0.001	0.001	0.37	-0.4 [-0.53, -0.26]	<0.001	0.003	0.24
pT231/T231	-0.47 [-0.56, -0.37]	<0.001	<0.001	0.23	-0.26 [-0.41, -0.12]	0.001	<0.001	0.16
MTBR-tau243	-0.65 [-0.74, -0.57]	<0.001	Ref.	0.41	-0.54 [-0.67, -0.42]	<0.001	Ref.	0.35
Aβ42/40	0.43 [0.34, 0.53]	<0.001	<0.001	0.20	0.28 [0.13, 0.42]	<0.001	0.067	0.17
Amyloid-PET	-0.2 [-0.25, -0.15]	<0.001	<0.001	0.22	-0.34 [-0.48, -0.21]	<0.001	0.001	0.21
Tau-PET	-0.72 [-0.79, -0.64]	<0.001	0.036	0.52	-0.64 [-0.74, -0.53]	<0.001	<0.001	0.50
**Aβ-positive participants**								
pT181/T181	-0.28 [-0.39, -0.17]	<0.001	<0.001	0.09	-0.11 [-0.29, 0.06]	0.208	<0.001	0.10
pT205/T205	-0.53 [-0.63, -0.43]	<0.001	0.510	0.29	-0.44 [-0.60, -0.28]	<0.001	0.222	0.28
pT217/T217	-0.48 [-0.59, -0.38]	<0.001	0.002	0.24	-0.36 [-0.52, -0.19]	<0.001	0.001	0.21

pT231/T231	-0.31 [-0.42, -0.20]	<0.001	<0.001	0.11	-0.14 [-0.31, 0.03]	0.113	<0.001	0.12
MTBR-tau243	-0.56 [-0.66, -0.46]	<0.001	Ref.	0.31	-0.54 [-0.69, -0.39]	<0.001	Ref.	0.37
Aβ42/40	0.23 [0.11, 0.34]	<0.001	<0.001	0.06	0.17 [0.00, 0.35]	0.053	0.139	0.12
Amyloid-PET	-0.14 [-0.21, -0.07]	<0.001	<0.001	0.09	-0.33 [-0.49, -0.16]	<0.001	0.003	0.19
Tau-PET	-0.63 [-0.72, -0.54]	<0.001	0.052	0.40	-0.63 [-0.76, -0.50]	<0.001	<0.001	0.50

Supplementary Table 6: Associations between AD-biomarkers and MMSE

Linear regression models were used to assess the associations between CSF biomarkers and MMSE for BioFINDER-2 and Knight ADRC participants adjusting for age, sex and education (BioFINDER-2: n=342, Knight ADRC: n=219, except for pT231/T231 in which n=184). Amyloid-positive participants (BioFINDER-2: n=261, Knight ADRC: n=136, except for pT231/T231 in which n=117) were selected based on CSF Aβ42/40 previously validated cut-offs (CSF Aβ42/40<0.08 in BioFINDER-2 and CSF Aβ42/40<0.0673 in Knight ADRC). P comparison (p comp.) was calculated to assess differences between the strongest association of CSF tau markers (Ref.) and each of the other biomarkers using bootstrapping (n=500) from adjusted β. Significant p comparison (<0.05) suggests weaker associations, except in the case of Tau-PET. Non-AD cases were excluded from BioFINDER-2 cohort for this analysis. Association p-values were based on two-sided tests and bootstrapping p-values from one-sided tests, all unadjusted for multiple comparisons.

Abbreviations: Aβ, amyloid; CI, confidence interval; CSF, cerebrospinal fluid; MMSE, Mini-Mental State Examination; MTBR, microtubule binding region; PET, positron emission tomography.

	BioFINDER-2		Knight ADRC			BioFINDER-2		Knight ADRC			BioFINDER-2		Knight ADRC	
Model	R^2	AICc	R^2	AICc	Model	R^2	AICc	R^2	AICc	Model	R^2	AICc	R^2	AICc
**Amyloid-PET (CL)**					**Tau-PET (SUVR)**					**MMSE**				
**All participants**														
Base	0.03	742.2	0.03	480.0	Base	0.01	1196.4	0.01	483.1	Base	0.02	970.3	0.10	468.5
pT181/T181	0.53	549.5	0.43	387.9	pT181/T181	0.43	961.8	0.15	456.1	pT181/T181	0.21	896.1	0.07	471.3
pT205/T205*†	0.51	563.1	0.46	381.0	pT205/T205*†	0.59	827.3	0.44	384.5	pT205/T205*†	0.41	797.0	0.26	433.0
pT217/T217*†	0.73	404.5	0.73	265.2	pT217/T217	0.60	813.1	0.36	409.6	pT217/T217	0.37	817.6	0.18	449.8
pT231/T231	0.61	503.5	0.45	381.7	pT231/T231	0.38	1002.1	0.20	447.8	pT231/T231	0.23	886.9	0.08	469.7
MTBR-tau243	0.42	605	0.34	412.7	MTBR-tau243*†	0.68	715.6	0.51	363.2	MTBR-tau243*†	0.42	790.3	0.30	423.0
Aβ42/40*†	0.59	514.8	0.61	325.2	Aβ42/40	0.30	1051.8	0.19	447.6	Aβ42/40	0.20	899.0	0.08	469.7
**Parsimonious**	0.77	370.2	0.73	261.7	**Parsimonious**	0.75	614.1	0.58	339.5	Parsimonious	0.48	754.6	0.34	415.2
-					-					**Tau-PET**	0.52	724.9	0.44	385.8
**Amyloid positive participants**														
Base	0.05	495.8	0.01	341.4	Base	0.01	817.4	0.00	343.4	Base	0.02	742.9	0.11	330.3
pT181/T181	0.35	429.0	0.21	314.4	pT181/T181	0.34	698.6	0.06	334.8	pT181/T181	0.10	718.6	0.02	339.9
pT205/T205*†	0.43	403.9	0.34	291.9	pT205/T205*†	0.56	585.7	0.42	277.6	pT205/T205*†	0.33	642.1	0.23	310.0
pT217/T217*†	0.56	359.4	0.49	262.3	pT217/T217	0.58	569.8	0.33	294.6	pT217/T217	0.28	658.0	0.15	322.5
pT231/T231*	0.43	405.6	0.27	304.3	pT231/T231	0.26	730.3	0.09	330.7	pT231/T231	0.12	711.3	0.02	339.6
MTBR-tau243	0.34	431.0	0.20	315.5	MTBR-tau243*†	0.66	506.4	0.51	256.8	MTBR-tau243*†	0.35	633.0	0.28	302.6
Aβ42/40*	0.27	449.0	0.19	316.3	Aβ42/40	0.17	765.6	0.09	330.5	Aβ42/40	0.07	725.8	0.02	339.6
**Parsimonious**	0.61	341.0	0.51	258.5	**Parsimonious**	0.74	433.9	0.58	239.9	Parsimonious	0.41	609.9	0.36	291.1
**-**					-					**Tau-PET**	0.46	587.9	0.44	273.9

Supplementary Table 7: Predicting AD-related continuous measure by CSF biomarkers

Linear regression models were used for predicting amyloid-PET (BioFINDER-2: n=256, Knight ADRC: n=184), tau-PET (BioFINDER-2: n=422, Knight ADRC: 184) and MMSE (BioFINDER-2: n=342, Knight ADRC: n=184). Base model included age and sex (and education for MMSE) as predictors. Parsimonious model was obtained with the optimal combination of CSF biomarkers and demographics (age and/or sex and/or education) assessed using a LASSO regression. Biomarkers included in the parsimonious models are depicted with * for BioFINDER-2 and with † for Knight ADRC. The other models used only individual CSF biomarkers as predictors. Tau-PET was used as predictor in an independent model for predicting MMSE as a comparison. Optimal models are shown in bold. Non-AD cases were excluded from BioFINDER-2 cohort for the cognition analyses. Amyloid-positive participants (BioFINDER-2: amyloid-PET: n=172, tau-PET: n=287, MMSE: n=261; Knight ADRC: n=117 for all cases) were selected based on CSF Aβ42/40 previously validated cut-offs (CSF Aβ42/40<0.08 in BioFINDER-2 and CSF Aβ42/40<0.0673 in Knight ADRC).

Abbreviations: Aβ, amyloid; AICc, corrected Akaike information criterion; CI, confidence interval; CL, Centiloids; CSF, cerebrospinal fluid; LASSO, least absolute shrinkage and selection operator; MMSE, Mini-Mental State Examination; MTBR, microtubule binding region; PET, positron emission tomography.
