# Supplementary material for: CSF MTBR-tau243 is a specific biomarker of tau tangle pathology in Alzheimer’s disease
Source: Nat Med. 2023 Jul 13;29(8):1954–63. doi: 10.1038/s41591-023-02443-z (PMC10427417; doi:10.1038/s41591-023-02443-z)
Supplement: Supplementary file 2 — Reporting summary [file 41591_2023_2443_MOESM2_ESM.pdf]

nature portfolio

Corresponding author(s): Randall J. Bateman, Oskar Hansson

Last updated by author(s): May 23, 2023

Reporting Summary

Nature Portfolio wishes to improve the reproducibility of the work that we publish. This form provides structure for consistency and transparency in reporting. For further information on Nature Portfolio policies, see our Editorial Policies and the Editorial Policy Checklist.

Statistics

For all statistical analyses, confirm that the following items are present in the figure legend, table legend, main text, or Methods section.

n/a	Confirmed	
☐	☒	The exact sample size (*n*) for each experimental group/condition, given as a discrete number and unit of measurement
☐	☒	A statement on whether measurements were taken from distinct samples or whether the same sample was measured repeatedly
☐	☒	The statistical test(s) used AND whether they are one- or two-sided<br>*Only common tests should be described solely by name; describe more complex techniques in the Methods section.*
☐	☒	A description of all covariates tested
☐	☒	A description of any assumptions or corrections, such as tests of normality and adjustment for multiple comparisons
☐	☒	A full description of the statistical parameters including central tendency (e.g. means) or other basic estimates (e.g. regression coefficient) AND variation (e.g. standard deviation) or associated estimates of uncertainty (e.g. confidence intervals)
☐	☒	For null hypothesis testing, the test statistic (e.g. *F*, *t*, *r*) with confidence intervals, effect sizes, degrees of freedom and *P* value noted<br>*Give P values as exact values whenever suitable.*
☒	☐	For Bayesian analysis, information on the choice of priors and Markov chain Monte Carlo settings
☒	☐	For hierarchical and complex designs, identification of the appropriate level for tests and full reporting of outcomes
☐	☒	Estimates of effect sizes (e.g. Cohen's *d*, Pearson's *r*), indicating how they were calculated

Our web collection on statistics for biologists contains articles on many of the points above.

Software and code

Policy information about availability of computer code

Data collection	NanoLC-MS/MS experiments were performed using nanoAcquity ultra-performance LC system (Waters) coupled to Orbitrap Tribrid Eclipse mass spectrometer (Thermo Scientific) operating in parallel reaction monitoring mode. MS transitions were extracted using Skyline v.22.2.0.255 (MacCoss lab, University of Washington).
Data analysis	Data were aggregated using Tableau v.2022.2.2 (Tableau Software) to calculate CSF biomarker levels. Differences in CSF biomarker levels were tested by diagnostic groups using ANCOVA adjusted for age and sex. Post-hoc analyses were performed using the Tuckey test. Linear regression models were used to assess the association between amyloid- and tau-PET (independent variable) and each of the CSF biomarkers (dependent variable), after adjusting for age and sex. For cognition, education was also used as covariate in the linear regression models. All standardized betas were compared to the highest for each outcome and cohort, by building a distribution of the betas' difference and using that to infer significance using a bootstrapping approach (n=500) with the boot package. Proportion of variation of CSF levels by amyloid and tau measures were assessed using linear regression models with both amyloid and tau as predictors, CSF levels as outcomes and age and sex as covariates. The partial R2 of amyloid and tau, raw and as a percentage of the total R2 of the model were calculated using the rsq package. This was used as a measure of proportion of variance explained by amyloid and tau. Next, prediction of amyloid and tau continuous measures was assessed with linear regression models, where amyloid- and tau-PET measures were used as outcomes in independent models and individual CSF biomarkers as predictors. A basic model was also created with only covariates (age and sex) as predictors. Additionally, a parsimonious model was constructed to optimally predict (highest accuracy with lower number of predictors) each of these measures, independently for each cohort. To this aim, LASSO regression models were used (glmnet package), initially including all CSF biomarkers and covariates. Only those predictors selected by the LASSO regression and with a significant contribution (p<0.1) in the model were finally included in the parsimonious model. Similar methods were used for predicting cognition (MMSE in the two cohorts and CDR in Knight ADRC) additionally including education as covariate. In these cases, the parsimonious model was compared to one including only tau-PET as predictor. F-tests were used to compare nested models (including the same subset of predictors). When comparing models with different

predictors we used the Vuong's test using the nonnest2 package. Finally, CSF longitudinal changes by baseline amyloid and tau status were assessed in the BioFINDER-2 cohort. Individual participant slopes were calculated using linear regression models to calculate rate of change differences and compare them between groups using a Kruskal-Wallis test. Further, we created group trajectories with linear mixed models using the lme4 package for visualization. Here, CSF biomarkers were used as outcome, interaction between time and baseline amyloid and tau status as predictor and age and sex main effects as covariates, using random intercepts and fixed time-slopes due to low number of time points. CSF and amyloid- and tau-PET measures were log-transformed in linear regression analyses. A two-sided P value <0.05 was considered statistically significant. R version 4.1.0 was used for all statistical analyses.

For manuscripts utilizing custom algorithms or software that are central to the research but not yet described in published literature, software must be made available to editors and reviewers. We strongly encourage code deposition in a community repository (e.g. GitHub). See the Nature Portfolio guidelines for submitting code & software for further information.

Data

Policy information about availability of data

All manuscripts must include a data availability statement. This statement should provide the following information, where applicable:

- Accession codes, unique identifiers, or web links for publicly available datasets
- A description of any restrictions on data availability
- For clinical datasets or third party data, please ensure that the statement adheres to our policy

The datasets generated and/or analyzed during the current study are available from the corresponding authors (R.J.B and O.H). We will share datasets within the restrictions of IRB ethics approvals, upon reasonable request. Pseudonymized data from the BioFINDER-2 will be shared by request from a qualified academic investigator for the sole purpose of replicating procedures and results presented in the article and as long as data transfer is in agreement with EU legislation on the general data protection regulation and decisions by the Ethical Review Board of Sweden and Region Skåne, which should be regulated in a material transfer agreement. Knight ADRC data are available to qualified investigators who have a proposal approved by an institutional committee (https://knightadrc.wustl.edu/Research/ResourceRequest.htm) that meets monthly. The study must be approved by an institutional review board to ensure ethical research practices and investigators must agree to the terms and conditions of the data use agreement, which includes not distributing the data without permission.

Human research participants

Policy information about studies involving human research participants and Sex and Gender in Research.

Reporting on sex and gender	Sex and race were self-identified.
Population characteristics	The BioFINDER-2 cohort included 448 individuals, the majority of whom had cognitive impairment (281, 63%): 81 cognitively unimpaired Amyloid negative (CU-), 79 cognitively unimpaired Amyloid positive (CU+), 90 Amyloid positive with mild cognitive impairment (MCI+), 102 Amyloid positive with AD dementia (AD+) and 96 with other dementias (non-AD). The average age was 70.9 ± 8.4 years (mean ± standard deviation), 221 (49.3%) were women, and 258 (57.6%) were APOE ε4 carriers. The Knight ADRC cohort included 219 individuals, most of whom were cognitively unimpaired (171, 78%): 83 CU-, 88 CU+, 35 very mild AD, and 13 AD+. The average age was 71.2 ± 6.6 years, 112 (51.1%) were women, and 96 (43.8%) were APOE ε4 carriers.
Recruitment	Participants were included from two cohorts: the Swedish BioFINDER-2 (NCT03174938) at Lund University (Lund, Sweden), and the Knight ADRC from Washington University (St Louis, MO, USA). BioFINDER-2 cohort included cognitively unimpaired participants (recruited as cognitively normal controls or as subjective cognitive decline [SCD] patients), patients with MCI, AD dementia patients and patients with a non-AD neurodegenerative disease. Participants were recruited at Skåne University Hospital and the Hospital of Ängelholm in Sweden. Details on recruitment, exclusion and inclusion criteria have been presented before (reference 11). All participants underwent lumbar puncture at baseline and at the follow-up after two years for CSF sampling. Participants underwent cognitive testing, including MMSE. The Knight ADRC cohort consisted of community-dwelling volunteers enrolled in studies of memory and aging at Washington University in St. Louis. All Knight ADRC participants underwent a comprehensive clinical assessment that included a detailed interview of a collateral source, a neurological examination of the participant, the Clinical Dementia Rating (CDR) and the MMSE. Individuals with a CDR of 0.5 or greater were considered to have a dementia syndrome and the probable aetiology of the dementia syndrome was formulated by clinicians based on clinical features in accordance with standard criteria and methods.
Ethics oversight	All participants in the Swedish BioFINDER-2 and the Knight ADRC cohorts gave written informed consent and ethical approvals were granted by the Regional Ethical Committee in Lund, Sweden and the Washington University Human Research Protection Office, respectively.

Note that full information on the approval of the study protocol must also be provided in the manuscript.

Field-specific reporting

Please select the one below that is the best fit for your research. If you are not sure, read the appropriate sections before making your selection.

☒ Life sciences ☐ Behavioural & social sciences ☐ Ecological, evolutionary & environmental sciences

For a reference copy of the document with all sections, see nature.com/documents/nr-reporting-summary-flat.pdf

Life sciences study design

All studies must disclose on these points even when the disclosure is negative.

Sample size	No statistical methods were used to pre-determine sample sizes but our sample sizes are similar or larger to those used for similar studies. (e.g., Mila-Aloma, M., et al. Nat Med 28, 1797-1801 (2022) and Barthelemy, N.R., et al. Nat Med 26, 398-407 (2020))
Data exclusions	No data points were excluded from analyses; outliers were not removed.
Replication	We replicated our key findings in two large independent cohorts (BioFINDER-2 and Knight ADRC) with significant differences in demographics and using different PET tracers.
Randomization	Samples were randomized by groups such as cognitively unimpaired participants, patients with MCI, AD dementia patients and patients with a non-AD neurodegenerative disease. All samples had a random code as an identifier and researchers who performed experiments were blinded towards the code when performing the analyses.
Blinding	All assays and data extraction steps were performed by operators blinded to any clinical or biomarker information.

Reporting for specific materials, systems and methods

We require information from authors about some types of materials, experimental systems and methods used in many studies. Here, indicate whether each material, system or method listed is relevant to your study. If you are not sure if a list item applies to your research, read the appropriate section before selecting a response.

Materials & experimental systems

n/a	Involved in the study	
☐	☒	Antibodies
☒	☐	Eukaryotic cell lines
☒	☐	Palaeontology and archaeology
☒	☐	Animals and other organisms
☐	☒	Clinical data
☒	☐	Dual use research of concern

Methods

n/a	Involved in the study	
☒	☐	ChIP-seq
☒	☐	Flow cytometry
☒	☐	MRI-based neuroimaging

Antibodies

Antibodies used	Tau1 (generated by Dr. Nicholas Kanaan) and HJ series (clone name: HJ8.5, HJ8.7, HJ32.11, and HJ34.8) antibodies (generated by Dr. David Holtzman) were used. For all antibodies, 3 mg/gram sepharose beads were generated. For Tau1 immunoprecipitation (IP), 1.1 ug antibody/sample was used. For HJ8.5 and HJ8.7 IP, 2.3 ug antibody/sample was used. For HJ32.11 and HJ34.8 IP, 11.25 ug antibody/sample was used.
Validation	Tau1, HJ8.5 and HJ8.7 were validated in the following studies.<br>1. Barthelemy NR, Toth B, Manser PT, et al. Site-Specific Cerebrospinal Fluid Tau Hyperphosphorylation in Response to Alzheimer's Disease Brain Pathology: Not All Tau Phospho-Sites are Hyperphosphorylated. Journal of Alzheimer's disease : JAD 2022; 85(1): 415-29.<br>2. Sato C, Barthelemy NR, Mawuenyega KG, et al. Tau Kinetics in Neurons and the Human Central Nervous System. Neuron 2018; 98(4): 861-4.<br>HJ32.11 and HJ34.8 were newly generated antibodies and we confirmed that immunoprecipitation procedures using these antibodies worked well by the two replicate cohorts analyses.

Clinical data

Policy information about clinical studies

All manuscripts should comply with the ICMJE guidelines for publication of clinical research and a completed CONSORT checklist must be included with all submissions.

Clinical trial registration	BioFINDER-2 (NCT03174938)<br>Knight ADRC (N/A--the study is NOT a clinical trial)
Study protocol	BioFINDER-2 (NCT03174938, https://clinicaltrials.gov/ct2/show/NCT03174938)<br>Knight ADRC (N/A--the study is NOT a clinical trial)
Data collection	Data was obtained from CSF collected from participants in the BioFINDER-2 cohort (NCT03174938) including cognitively unimpaired participants, patients with MCI, AD dementia patients and patients with a non-AD neurodegenerative disease. The participants from the prospective Swedish BioFINDER-2 study were recruited at the Memory and Neurology clinics of Skåne University Hospital and the

Memory clinic of Ängelholm's Hospital in Sweden (dates of enrollment, February 2017-April 2021). Participants in the Knight ADRC cohort were community-dwelling volunteers enrolled in studies of memory and aging.

Outcomes

Main outcomes of the study included amyloid- and tau-PET, as well as cognition, assessed with MMSE. Amyloid-PET was measured in a neocortical meta-ROI using cerebellar grey as a reference region. Tau-PET SUVRs were calculated using the inferior cerebellum cortex as reference region and binding from a temporal meta-ROI were used for main analyses. Tau-PET SUVRs in Braak I, Braak III-IV and Braak V-VI were also assessed as secondary outcomes.
